# Optical coherence tomography angiography analysis methods: a systematic review and meta-analysis

**DOI:** 10.1038/s41598-024-54306-3

**Published:** 2024-04-26

**Authors:** Ella Courtie, James Robert Moore Kirkpatrick, Matthew Taylor, Livia Faes, Xiaoxuan Liu, Ann Logan, Tonny Veenith, Alastair K. Denniston, Richard J. Blanch

**Affiliations:** 1 Neuroscience and Ophthalmology Research Group, University of Birmingham, Birmingham, UK; 2 Department of Ophthalmology, Queen Elizabeth Hospital Birmingham, University Hospitals Birmingham NHS Foundation Trust, Birmingham, West Midlands UK; 3 Surgical Reconstruction and Microbiology Research Centre, University Hospitals Birmingham NHS Foundation Trust, Birmingham, UK; 4 1 Armoured Medical Regiment, British Army, Bhurtpore Barracks, Tidworth, UK; 5 University Hospitals Birmingham NHS Foundation Trust, Birmingham, UK; 6 University of Birmingham, Birmingham, UK; 7 Birmingham Women’s and Children’s NHS Foundation Trust, Birmingham, UK; 8 NIHR Biomedical Research Centre at Moorfields Eye Hospital NHS Foundation Trust, UCL Institute of Ophthalmology, London, UK; 9 NIHR Birmingham Biomedical Research Centre, University Hospitals Birmingham NHSFT, Birmingham, UK; 10 Axolotl Consulting Ltd., Droitwich, Worcestershire UK; 11 Division of Biomedical Sciences, Warwick Medical School, University of Warwick, Coventry, UK; 12 Critical Care Unit, Queen Elizabeth Hospital Birmingham, University Hospitals Birmingham NHS Foundation Trust, Birmingham, UK; 13 Department of Trauma Sciences, University of Birmingham, Birmingham, UK; 14 Academic Department of Military Surgery and Trauma, Royal Centre for Defence Medicine, Birmingham, UK

**Keywords:** Predictive markers, Medical imaging

## Abstract

Optical coherence tomography angiography (OCTA) is widely used for non-invasive retinal vascular imaging, but the OCTA methods used to assess retinal perfusion vary. We evaluated the different methods used to assess retinal perfusion between OCTA studies. MEDLINE and Embase were searched from 2014 to August 2021. We included prospective studies including ≥ 50 participants using OCTA to assess retinal perfusion in either global retinal or systemic disorders. Risk of bias was assessed using the National Institute of Health quality assessment tool for observational cohort and cross-sectional studies. Heterogeneity of data was assessed by Q statistics, Chi-square test, and I^2^ index. Of the 5974 studies identified, 191 studies were included in this evaluation. The selected studies employed seven OCTA devices, six macula volume dimensions, four macula subregions, nine perfusion analyses, and five vessel layer definitions, totalling 197 distinct methods of assessing macula perfusion and over 7000 possible combinations. Meta-analysis was performed on 88 studies reporting vessel density and foveal avascular zone area, showing lower retinal perfusion in patients with diabetes mellitus than in healthy controls, but with high heterogeneity. Heterogeneity was lowest and reported vascular effects strongest in superficial capillary plexus assessments. Systematic review of OCTA studies revealed massive heterogeneity in the methods employed to assess retinal perfusion, supporting calls for standardisation of methodology.

## Introduction

Optical coherence tomography (OCT) is a non-invasive, non-contact imaging modality which provides high resolution, cross-sectional images of the retina and is ubiquitous in ophthalmology practice to diagnose and monitor retinal disorders^1^. OCT angiography (OCTA) uses moving red blood cells in the retinal vasculature as an intrinsic contrast agent to generate 3-dimensional images of retinal and choroidal blood flow^2,3^. OCTA is widely used to evaluate retinal perfusion in retinal and systemic disorders^4^, and demonstrates microvascular impairment in disorders such as diabetes mellitus^5^, uveitis^6^, age-related macular degeneration^7^, atrial fibrillation^8^, haemorrhagic shock^9,10^, and systemic hypertensive crisis^11^. As OCTA is fast, cheap, and does not risk systemic reactions (as fundus fluorescein angiography (FFA) or indocyanine green angiography do), its use is fast becoming widespread in research and clinical practice. OCTA is now used alongside OCT and FFA in the diagnosis and management of retinal diseases^12^.

Many OCTA platforms use proprietary algorithms to estimate and visualise retinal perfusion^13,14^. However, as different OCTA devices use different algorithms, comparisons of results between studies are constrained^13^. Further, quantitative metrics derived from the OCTA signal and images lack consistent methodology^15^, also limiting comparison validity^16^. The raw signal may be used to derive limited scaled flow information^15^, and additional processing before image analysis includes thresholding to create binary images from grayscale^17^, and skeletonization to display vessels as one-pixel width tracings^18^. The most commonly calculated perfusion metrics from binarised and skeletonised images are^17,19^:

1. Vessel density (VD)—the total area of perfused vasculature per unit area in a region of measurement (sometimes reported as “perfusion density”).
2. Vessel length density (VLD)—the total length of perfused vessels divided by the total number of pixels in the given area on the skeletonised image.
3. Fractal dimension (FD)—a mathematical parameter describing the complexity of a biological structure, usually applied to skeletonised images^20^.
4. Foveal avascular zone (FAZ) measurements (Supplementary Fig. 1)—a change in FAZ measurements (e.g. area and perimeter) from baseline suggests altered blood flow^21^.

A scoping search on the National Library of Medicine PubMed (including Medical Literature Analysis and Retrieval System Online—MEDLINE) found no existing systematic reviews or meta-analyses comparing methods of quantitative OCTA analysis. We therefore conducted a systematic review and meta-analysis with the aim of assessing which OCTA perfusion analysis method most sensitively detects pathological change between patients with disorders affecting retinal perfusion and control patients with normal retinal perfusion. Our secondary aim was to look at the stability of OCTA imaging by identifying papers that studied the test–retest variability of OCTA.

## Methods

This systematic review was performed following the recommendations of the Preferred Reporting Items for Systematic Reviews and Meta-Analysis Protocols (PRISMA) statement^22^.

### Inclusion criteria

Full inclusion and exclusion criteria are provided in Supplementary Table 1. We initially sought to investigate the sensitivity and stability of OCTA imaging, therefore we planned to include both studies comparing findings in normal patients with pathology and studies that included patients having repeated OCTA scans over time with or without a control group.

Prospective studies involving ≥ 50 participants were included where OCTA had been used to investigate changes to macula perfusion caused by either retinal or systemic disorders, using any one of the following analysis metrics (either on binarized or skeletonised images): VD, skeletonised VD (SVD), VLD, FD, skeletonised FD (SFD), capillary density index, FAZ measurements and; where agreement between repeated OCTA images was assessed by intra-class correlation coefficient. Included studies were limited to those with a sample size of at least 50 participants to minimise selection bias from the inclusion of small and selective case series. The year of publication was limited from 2014 to August 2021, as the clinical application of OCTA was first described in 2014^23^. Only studies looking at foveal, parafoveal, and whole areas of the macula were included.

Papers published in medical journals and written in English were included—conference abstracts and papers written in languages other than English were excluded.

### Exclusion criteria

We excluded studies investigating retinal disorders which cause focal anatomical change (e.g., age-related macular degeneration) or studies that only investigated perfusion in the choroid, choriocapillaris, or peripapillary region. Retrospective studies and studies that did not specify which region of the macula was analysed were excluded.

### Search strategy

MEDLINE and Embase were searched using OVID. The applied search strategy is in Supplementary Fig. 2.

### Risk of bias assessment

Two authors independently assessed the potential bias in the prospective studies using the National Institutes of Health (NIH) quality assessment tool for observational cohort and cross-sectional studies^24^. A consensus was then reached between the two authors to create the risk of bias table (Supplementary Table 3).

### Data extraction

**Figure 1:**
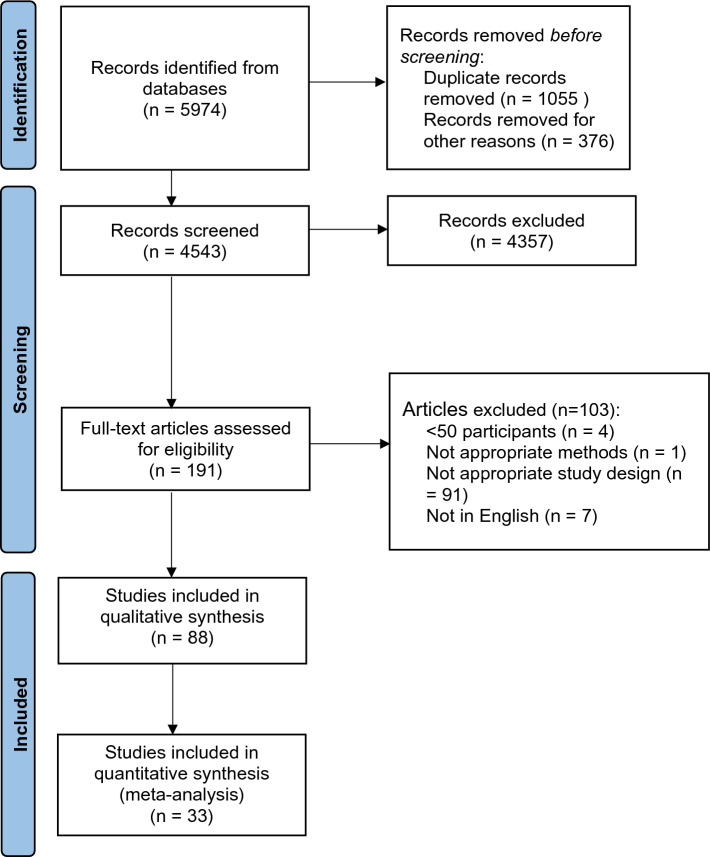
Systematic review and meta-analysis study flowchart.

Retinal perfusion was compared between healthy control patients and those with defined disease states. Two independent reviewers individually reviewed all titles and abstracts retrieved from the initial search. Duplicates were removed and each reviewer decided on the study’s inclusion based on the title and abstract. Disagreements between reviewers on a paper’s eligibility were resolved by discussion, involving the senior author (RJB) if a decision could not be reached. Reference management software was used to aid the screening process as per the PRISMA flow diagram (Fig. 1). Data were extracted by two reviewers working independently, with disagreements resolved by discussion. The following variables were recorded: study information (first author, year of publication, country location of study, study design), participant information (total number of eyes, total number of patients, sex, mean age), OCTA device and imaging information (instrument manufacturer, number of a-scans, scan size), and OCTA analysis information (vessel layer, macular region, analysis metric mean and standard deviation).

The outcome data (mean and standard deviation) collected included: percentage VD (VD%), SVD, VLD, FD, SFD, FAZ area, FAZ perimeter, FAZ acircularity ratio, and FAZ acircularity index. If unpublished information was required, the corresponding author of the study was contacted. If no response was received within one-month of contact, analysis proceeded based on published data. Only VD data given as a percentage and FAZ area presented as mm^2^ were included in the study characteristics table.

### Statistical methods for the effect of diabetic retinopathy on retinal perfusion

To combine measurements of the continuous variables VD and FAZ, and estimate a value for overall common and random effects, inverse variance weighting was used for pooling. When comparisons were made between pooled standardised mean differences for different sub-analyses, statistical differences were assessed using a Z test, with *p* < 0.05 considered statistically significant. An overall standardised mean difference was calculated using the random effects models. A funnel plot was used to detect publication and location bias in the selection of included trials according to the method of Egger et al.^25^. The R statistical software (Version 4.1.1) (R Foundation for Statistical Computing, Vienna, Austria; see http://www.r-project.org) and its meta package (http://cran.r-project.org/web/package/meta) were used for these analyses.

### Statistical methods for evaluating the effect of analysis methods on the assessment of retinal perfusion in diabetes without diabetic retinopathy or with mild non-proliferative retinopathy

Meta-analyses were performed on studies investigating diabetes mellitus with no diabetic retinopathy or with mild, non-proliferative diabetic retinopathy (the early stage of diabetic retinopathy in which symptoms are mild or non-existent), using Review Manager 5 (RevMan Version 5.4. The Cochrane Collaboration, 2020). Statistical heterogeneity between studies was tested for using the Q-statistic (tests the null hypothesis that all studies share the same common effect) and heterogeneity was quantified using the I^2^ measure of study heterogeneity (percentage of total variation across studies that is due to true heterogeneity rather than chance). A random effects model was used to address the issue of high levels of heterogeneity of results between studies.

## Results

After removing duplicates, electronic searches retrieved 4543 records, of which 191 studies were included, and 88 eligible for qualitative analysis. A PRISMA flow diagram of search results is presented in Fig. 1. Excluded studies are presented in the Supplementary Table 2.

### Characteristics of included studies

Study characteristics are presented in Tables 1 and 2. Of the 88 studies included, 78 were cross-sectional, six were longitudinal cohort studies, and four were case–control studies. Five papers met the original inclusion criteria but were not presented in the study characteristics table, as they did not include VD or FAZ data, instead using VLD or FD. Only summary data defined as VD or FAZ is presented as reporting of other analysis methods was too heterogenous. Some studies did not specify which macula region was analysed for VD but did include FAZ data. In these instances, the paper was included but VD data were excluded. While the baseline data were presented from five longitudinal studies that included patients having repeated OCTA scans over time, no studies reported test–retest variability.

**Table 1:** Study characteristics of studies included looking at patients with diabetes.

References	Manufacturer	Diagnosis included	Mean age (years)	Study Design	Number of eyes (number of patients)	Number of males/females	Scan size (mm)	Metric	Vessel layer	Region	Mean	SD
Agra et al.^38^	Optovue, Avanti	DM NDR	60.00	Cross-sec	60 (60)	19/41	6 × 6	VD	SCP	Whole	52.40	3.20
		Healthy controls	60.00								51.20	4.80
		DM NDR						VD	DCP	Whole	54.30	3.20
		Healthy controls									53.10	4.40
		DM NDR						VD	SCP	Para-foveal	55.40	3.80
		Healthy controls									55.40	4.10
		DM NDR						VD	DCP	Para-foveal	58.10	3.60
		Healthy controls									57.50	4.30
		DM NDR						VD	SCP	Fovea	28.30	5.90
		Healthy controls									27.70	5.00
		DM NDR						VD	DCP	Fovea	28.30	6.40
		Healthy controls									28.20	5.80
		DM NDR						FAZ area	SCP	Whole	0.40	0.10
		Healthy controls									0.40	0.10
Carnevali et al.^39^	Zeiss, Cirrus HD-OCT	DM NDR	22.0	Cross-sec	50 (50)	30/20	3 × 3	FAZ area	SCP	Fovea	0.22	0.10
		Healthy controls	23.0								0.25	0.10
		DM NDR						FAZ area	DCP	Fovea	0.75	0.20
		Healthy controls									0.76	0.23
Choi et al.^40^	Zeiss, Cirrus HD-OCT	DM NDR	62.5	Cross-sec	103 (103)	51/52	6 × 6	FAZ area	SCP	Fovea	0.37	0.13
		Healthy controls	62.9								0.29	0.11
		DM NDR						FAZ area	DCP	Fovea	0.75	0.19
		Healthy controls									0.71	0.23
Cinar et al.^41^	Optovue, Avanti	DM NDR	49.5	Cross-sec	96 (96)	48/48	6 × 6	VD	SCP	Para-foveal	55.11	1.11
		Healthy controls	48.5								55.87	1.35
		DM NDR						VD	DCP	Para-foveal	54.21	1.02
		Healthy controls									57.64	0.43
		DM NDR						VD	SCP	Fovea	35.42	1.33
		Healthy controls									36.00	1.88
		DM NDR						VD	DCP	Fovea	36.01	0.54
		Healthy controls									37.67	0.54
		DM NDR						FAZ area	SCP	Fovea	0.35	0.01
		Healthy controls									0.31	0.01
		DM NDR						FAZ area	DCP	Fovea	0.36	0.01
		Healthy controls									0.32	0.02
De Carlo et al.^42^	Optovue, Avanti	DM NDR	60.0	Cross-sec	89 (61)	29/32	3 × 3	FAZ area	Full	Fovea	0.35	0.10
		Healthy controls	54.0								0.29	0.14
Demir et al.^43^	Optovue, Avanti	DM NDR	12.30	Cross-sec	194 (97)	44/53	3 × 3	VD	SCP	Para-foveal	50.10	3.20
		Healthy controls	11.7								50.70	2.50
		DM NDR						VD	DCP	Para-foveal	54.60	3.50
		Healthy controls									55.10	3.50
		DM NDR						VD	SCP	Fovea	18.40	5.70
		Healthy controls									18.50	5.80
		DM NDR						VD	DCP	Fovea	34.50	6.30
		Healthy controls									34.50	7.20
Durbin et al.^18^	Zeiss, Cirrus HD-OCT	DM NDR/mild NPDR	64.9	Cross-sec	100 (51)	27/24	3 × 3	FAZ area	SRL	Fovea	0.26	0.10
		Healthy controls	64.0								0.25	0.10
Furino et al.^44^	Topcon, Triton	DM NDR	58.3	Cross-sec	164 (82)	Unknown	3 × 3	VD	SCP	Un-known	14.22	1.40
		Healthy controls	56.4								14.24	1.39
		DM NDR						VD	DCP	Un-known	17.33	1.67
		Healthy controls									17.95	1.58
		DM NDR						FAZ area	SCP	Fovea	2.98	1.26
		Healthy controls									2.48	1.16
		DM NDR						FAZ area	DCP	Fovea	1.18	1.16
		Healthy controls									1.01	0.97
		DM NDR					4.5 × 4.5	VD	SCP	Un-known	14.18	1.38
		Healthy controls									14.48	1.32
		DM NDR						VD	DCP	Un-known	16.28	2.62
		Healthy controls									17.00	1.89
Golebiewska et al.^45^	Optovue, Avanti	DM NDR	15.3	Cross-sec	248 (130)	Unknown	3 × 3	VD	SCP	Whole	51.98	2.43
		Healthy controls	13.6								52.45	2.74
		DM NDR						VD	DCP	Whole	58.57	1.95
		Healthy controls									58.57	5.03
		DM NDR						VD	SCP	Para-foveal	53.80	2.54
		Healthy controls									54.41	2.62
		DM NDR						VD	DCP	Para-foveal	61.28	2.10
		Healthy controls									No Data	No Data
		DM NDR						VD	SCP	Fovea	32.51	5.26
		Healthy controls									32.48	5.33
		DM NDR						VD	DCP	Fovea	32.37	6.17
		Healthy controls									31.75	3.96
		DM NDR						VD	SCP	Whole	0.23	0.10
		Healthy controls									0.24	0.08
Inanc et al.^46^	Optovue, Avanti	DM NDR	13.8	Cross-sec	117 (117)	47/70	6 × 6	VD	SCP	Whole	50.43	3.14
		Healthy controls	14.1								51.16	2.82
		DM NDR						VD	DCP	Whole	52.32	5.24
		Healthy controls									53.36	4.66
		DM NDR						VD	SCP	Para-foveal	52.96	3.44
		Healthy controls									54.18	2.78
		DM NDR						VD	DCP	Para-foveal	56.77	4.05
		Healthy controls									57.64	3.50
		DM NDR						VD	SCP	Fovea	20.50	5.71
		Healthy controls									20.72	6.14
		DM NDR						VD	DCP	Fovea	38.29	6.55
		Healthy controls									39.24	6.66
		DM NDR						FAZ area	Full	Fovea	0.28	0.11
		Healthy controls									0.27	0.13
Kara et al.^47^	Optovue, Avanti	DM NDR	13.8	Cross-sec	238 (119)	46/73	6 × 6	VD	SCP	Whole	50.42	2.20
		Healthy controls	13.4								51.69	2.12
		DM NDR						VD	DCP	Whole	53.79	5.00
		Healthy controls									56.11	4.76
		DM NDR						VD	SCP	Para-foveal	52.98	3.28
		Healthy controls									53.94	3.01
		DM NDR						VD	DCP	Para-foveal	57.10	3.89
		Healthy controls									58.85	3.78
		DM NDR						VD	SCP	Fovea	21.05	6.88
		Healthy controls									23.13	6.90
		DM NDR						VD	DCP	Fovea	37.94	7.55
		Healthy controls									40.17	7.59
		DM NDR						FAZ area	Full	Fovea	0.28	0.10
		Healthy controls									0.27	0.11
Meshi et al.^48^	Optovue, Avanti	DM NDR	58.5	Case control	105 (66)	30/36	3 × 3	VD	SCP	Un-known	44.61	5.90
		Healthy controls	58.9								44.75	4.90
		DM NDR						VD	DCP	Un-known	52.74	6.30
		Healthy controls									55.45	4.30
		DM NDR						FAZ area	SCP	Fovea	0.251	0.09
		Healthy controls									0.261	0.11
		DM NDR						FAZ area	DCP	Fovea	0.311	0.09
		Healthy controls									0.321	0.11
Li T et al.^49^	Optovue, Avanti	DM NDR	11.1	Cross-sec	Unknown (91)	39/52	6 × 6	VD	SCP	Para-foveal	18.56	1.15
		Healthy controls	10.2								19.18	0.46
		DM NDR						VD	SCP	Fovea	11.24	3.30
		Healthy controls									11.80	2.54
Sacconi et al.^50^	Zeiss, PLEX Elite 9000	DM NDR	21.0	Cross-sec	66 (66)	34/32	3 × 3	FAZ area	SCP	Fovea	0.235	0.072
		Healthy controls	22.0								0.199	0.100
		DM NDR						FAZ area	DCP	Fovea	0.670	0.178
		Healthy controls									0.620	0.257
Vujosevic et al.^51^	Topcon, Triton	DM NDR	57.4	Cross-sec	60 (60)	Unknown	3 × 3	FAZ area	SCP	Fovea	0.359	0.120
		Healthy controls	44.4		60 (60)						0.286	0.137
		DM NDR						FAZ area	DCP	Fovea	0.497	0.150
		Healthy controls									0.364	0.142
Yang et al.^52^	Optovue, Avanti	DM NDR	68.6	Cross-sec	372 (259)	146/226	3 × 3	VD	SRL	Whole	42.40	5.09
		Healthy controls	66.8								45.04	4.32
		DM NDR						VD	SRL	Para-foveal	45.35	5.41
		Healthy controls									47.91	4.49
		DM NDR						VD	SRL	Fovea	14.42	5.95
		Healthy controls									15.52	6.55
		DM NDR						VD	DRL	Whole	50.16	3.99
		Healthy controls									49.43	3.22
		DM NDR						VD	DRL	Para-foveal	52.75	4.05
		Healthy controls									51.38	5.42
		DM NDR					6 × 6	VD	SRL	Whole	45.93	4.61
		Healthy controls									48.46	4.03
		DM NDR						VD	SRL	Para-foveal	46.53	4.78
		Healthy controls									49.06	4.36
		DM NDR						VD	SRL	Fovea	16.58	7.48
		Healthy controls									17.58	7.25
		DM NDR					3 × 3	FAZ area	SRL	Fovea	0.42	0.75
		Healthy controls									0.34	0.13
Zeng et al.^53^	Optovue, Avanti	DM NDR	58.8	Cross-sec	128 (128)	69/59	6 × 6	VD	SCP	Para-foveal	49.97	4.45
		Healthy controls	55.2								53.47	4.31
		DM NDR						VD	DCP	Para-foveal	52.70	4.51
		Healthy controls									55.99	4.09
Forte et al.^54^	Topcon, Triton	T1DM NDR	34.5	Cross-sec	29 (17)		3 × 3	FAZ area	SCP	Fovea	0.283	0.08
		T2DM NDR	48.8		32 (17)						0.296	0.12
		Healthy controls	41.8		43 (23)						0.218	0.07
		T1DM NDR						FAZ area	DCP	Fovea	0.321	0.01
		T2DM NDR									0.353	0.15
		Healthy controls									0.252	0.08
Bhanushali et al.^55^	Optovue, Avanti	DM mild NPDR	64.3	Cross-sec	269 (153)	87/66	3 × 3	VD	SRVP	Un-known	39.20	1.21
		DM moderate NPDR	61.1								40.10	0.58
		DM severe NPDR	59.6								38.50	0.76
		DM PDR	59.1								38.90	1.38
		Healthy controls	Unknown								49.70	0.55
		DM mild NPDR						VD	DRVP	Un-known	39.70	1.57
		DM moderate NPDR									40.20	0.53
		DM severe NPDR									39.40	0.68
		DM PDR									39.20	0.94
		Healthy controls									53.10	0.73
		DM mild NPDR						FAZ area	SRVP	Fovea	0.46	0.03
		DM moderate NPDR									0.45	0.01
		DM severe NPDR									0.46	0.02
		DM PDR									0.47	0.02
		Healthy controls									0.30	0.01
Bontzos et al.^56^	Optovue, Avanti	DM NDR	53.1	Cross-sec	162 (162)	85/77	6 × 6	VD	SCP	Fovea	32.82	3.25
		DM mild NPDR	55.7								30.21	4.19
		Healthy controls	48.2								33.60	3.52
		DM NDR						VD	DCP	Fovea	48.67	4.41
		DM mild NPDR									41.55	4.37
		Healthy controls									50.22	3.48
Cao et al.^57^	Optovue, Avanti	DM mild NPDR	57.4	Cross-sec	138 (138)	66/72	6 × 6	VD	SCP	Whole	51.34	4.09
		Healthy controls	53.7								55.72	2.43
		DM mild NPDR						VD	DCP	Whole	57.66	5.73
		Healthy controls									62.10	2.11
		DM mild NPDR						FAZ area	SCP	Fovea	0.32	0.18
		Healthy controls									0.35	0.09
		DM mild NPDR						VD	SCP	Para-foveal	53.99	4.72
		Healthy controls									58.69	2.12
		DM mild NPDR						VD	DCP	Para-foveal	62.01	5.17
		Healthy controls									65.25	2.01
Ciloglu et al.^58^	Optovue, Avanti	DM mild NPDR	56.6	Cross-sec	94 (94)	50/44	3 × 3	VD	SCP	Para-foveal	45.43	0.56
		Healthy controls	54.1								52.17	0.58
		DM mild NPDR						VD	DCP	Para-foveal	52.82	0.85
		Healthy controls									60.68	0.90
		DM mild NPDR						VD	SCP	Fovea	29.45	0.76
		Healthy controls									34.86	0.75
		DM mild NPDR						VD	DCP	Fovea	24.85	1.08
		Healthy controls									33.47	0.56
		DM mild NPDR						FAZ area	SCP	Fovea	0.44	0.05
		Healthy controls									0.25	0.02
		DM mild NPDR						FAZ area	DCP	Fovea	0.73	0.06
		Healthy controls									0.34	0.02
Czako et al.^59^	Optovue, Avanti	DM mild NPDR	58.5	Cross-sec	194 (97)	58/39	3 × 3	VD	SCP	Whole	47.04	3.24
		DM NDR	58.5								48.94	3.33
		Healthy controls	58.2								51.16	3.28
		DM mild NPDR						VD	SCP	Para-foveal	48.47	3.93
		DM NDR									51.26	3.72
		Healthy controls									53.25	3.36
		DM mild NPDR						FAZ area	SCP	Fovea	0.31	0.06
		DM NDR									0.29	0.07
		Healthy controls									0.28	0.06
Kim et al.^60^	Topcon, Triton	DM PDR	65.9	Cohort	523 (Unknown)	156/367	3 × 3	VD	SCP	Un-known	34.77	0.70
		DM severe NPDR	64.5								35.27	0.84
		DM moderate NPDR	63.3								35.14	0.79
		DM mild NPDR	67.1								35.73	0.85
		DM NDR	65.7								35.90	0.81
		Healthy controls	65.2								35.95	0.59
		DM PDR						VD	DCP	Un-known	23.05	3.07
		DM severe NPDR									24.18	3.76
		DM moderate NPDR									24.10	4.51
		DM mild NPDR									24.27	8.34
		DM NDR									24.20	4.38
		Healthy controls									24.87	4.35
		DM PDR						FAZ area	SCP	Fovea	0.50	0.17
		DM severe NPDR									0.47	0.10
		DM moderate NPDR									0.42	0.11
		DM mild NPDR									0.41	0.10
		DM NDR									0.42	0.10
		Healthy controls									0.40	0.13
		DM PDR						FAZ area	DCP	Fovea	0.64	0.19
		DM severe NPDR									0.60	0.15
		DM moderate NPDR									0.63	0.21
		DM mild NPDR									0.60	0.22
		DM NDR									0.55	0.18
		Healthy controls									0.52	0.14
Koçer et al.^61^	Optovue, Avanti	DM PDR	56.6	Cross-sec	128 (128)	52/76	6 × 6	VD	SCP	Whole	45.30	3.70
		DM severe NPDR	56.8								46.90	2.60
		DM moderate NPDR	53.9								47.00	2.90
		DM mild NPDR	52.2								46.30	2.50
		DM NDR	53.8								48.80	4.40
		Healthy controls	53.9								49.90	3.20
		DM PDR						VD	SCP	Para-foveal	45.10	4.60
		DM severe NPDR									47.40	2.80
		DM moderate NPDR									46.90	2.90
		DM mild NPDR									46.60	3.60
		DM NDR									49.90	5.20
		Healthy controls									52.00	4.20
		DM PDR						VD	SCP	Fovea	14.70	6.30
		DM severe NPDR									16.40	5.30
		DM moderate NPDR									17.90	7.20
		DM mild NPDR									16.50	8.60
		DM NDR									17.70	7.10
		Healthy controls									21.20	6.00
		DM PDR						VD	DCP	Whole	43.80	3.40
		DM severe NPDR									47.20	3.20
		DM moderate NPDR									45.90	4.40
		DM mild NPDR									46.70	5.20
		DM NDR									49.90	7.70
		Healthy controls									50.20	7.60
		DM PDR						VD	DCP	Para-foveal	47.70	3.50
		DM severe NPDR									50.40	3.10
		DM moderate NPDR									49.90	3.30
		DM mild NPDR									51.00	3.50
		DM NDR									54.20	4.80
		Healthy controls									55.70	3.80
		DM PDR						VD	DCP	Fovea	30.00	7.80
		DM severe NPDR									30.90	6.00
		DM moderate NPDR									30.80	5.90
		DM mild NPDR									29.40	10.40
		DM NDR									33.30	8.70
		Healthy controls									41.10	6.10
		DM PDR						FAZ area	SCP	Fovea	0.34	0.13
		DM severe NPDR									0.33	0.09
		DM moderate NPDR									0.33	0.08
		DM mild NPDR									0.36	0.15
		DM NDR									0.30	0.11
		Healthy controls									0.21	0.06
Li H et al.^62^	Optovue, Avanti	DM mild NPDR	57.0	Cross-sec	258 (132)	Unknown	3 × 3	VD	SCP	Whole	40.40	5.20
		DM NDR	53.0								44.40	4.10
		Healthy controls	53.0								46.50	2.60
		DM mild NPDR						VD	SCP	Para-foveal	42.80	5.50
		DM NDR									47.30	4.40
		Healthy controls									49.40	3.10
		DM mild NPDR						VD	DCP	Whole	45.30	4.50
		DM NDR									49.10	3.70
		Healthy controls									51.20	7.10
		DM mild NPDR						VD	DCP	Para-foveal	47.60	5.00
		DM NDR									51.70	3.90
		Healthy controls									53.80	7.40
Ryu et al.^63^	Optovue, Avanti	DM PDR	57.4	Cross-sec	190 (190)	103/87	3 × 3	VD	SCP	Para-foveal	39.88	4.82
		DM mild NPDR	58.7								43.97	4.18
		DM NDR	60.7								46.56	5.45
		Healthy controls	57.8								49.85	3.26
		DM PDR						VD	DCP	Para-foveal	44.40	4.31
		DM mild NPDR									46.93	4.20
		DM NDR									51.60	2.72
		Healthy controls									43.97	4.18
		DM PDR						FAZ area	SCP	Fovea	0.431	0.195
		DM mild NPDR									0.386	0.109
		DM NDR									0.307	0.101
		Healthy controls									0.327	0.09
		DM PDR						FAZ area	DCP	Fovea	0.369	0.193
		DM mild NPDR									0.293	0.083
		DM NDR									0.253	0.054
		Healthy controls									0.270	0.073
Shen et al.^64^	Optovue, Avanti	DM mild NPDR	56.4	Cross-sec	90 (90)	49/41	3 × 3	VD	SCP	Whole	47.82	4.62
		Healthy controls	52.9								54.10	2.10
		DM mild NPDR						VD	SCP	Para-foveal	49.30	5.12
		Healthy controls									56.60	2.19
		DM mild NPDR						VD	SCP	Fovea	28.38	5.57
		Healthy controls									34.48	5.98
Simonett et al.^65^	Optovue, Avanti	DM NDR/mild NPDR	42.3	Cross-sec	51 (51)	23/28	3 × 3	VD	SCP	Para-foveal	49.80	4.20
		Healthy controls	39.6								51.50	4.00
		DM NDR/mild NPDR						VD	DCP	Para-foveal	57.00	3.10
		Healthy controls									60.70	2.40
		DM NDR/mild NPDR						FAZ area	SCP	Fovea	0.26	0.12
		Healthy controls									0.26	0.11
		DM NDR/mild NPDR						FAZ area	DCP	Fovea	0.40	0.15
		Healthy controls									0.38	0.15
Somilleda-Ventura et al.^66^	Zeiss, Cirrus HD-OCT 5000	DM mild NPDR	56.7	Cross-sec	77 (52)	7/45	3 × 3	VD	SCP	Whole	18.45	1.73
		DM NDR	55.7								19.49	1.53
		Healthy controls	55.7								20.06	2.11
		DM mild NPDR						VD	SCP	Para-foveal	19.90	1.80
		DM NDR									20.78	1.52
		Healthy controls									21.11	2.29
		DM mild NPDR						VD	SCP	Fovea	7.00	2.07
		DM NDR									9.32	2.46
		Healthy controls									11.69	2.60
		DM mild NPDR						FAZ area	SCP	Fovea	0.38	0.10
		DM NDR									0.28	0.09
		Healthy controls									0.22	0.10
Buyuktepe et al.^67^	Optovue, Avanti	DM NPDR	50.8	Cross-sec	52 (52)		6 × 6	VD	SCP	Whole	47.53	3.33
		DM NDR	55.5		44 (44)						45.36	13.28
		Healthy controls	58.1		20 (20)						50.59	2.30
		DM NPDR						VD	SCP	Para-foveal	55.02	5.67
		DM NDR									50.29	4.36
		Healthy controls									52.76	2.47
		DM NPDR						VD	SCP	Fovea	19.13	6.19
		DM NDR									18.70	7.35
		Healthy controls									22.68	6.80
		DM NPDR						VD	DCP	Whole	46.94	4.29
		DM NDR									50.46	5.99
		Healthy controls									49.25	3.56
		DM NPDR						VD	DCP	Para-foveal	50.27	3.53
		DM NDR									54.76	4.43
		Healthy controls									53.56	2.73
		DM NPDR						VD	DCP	Fovea	32.47	6.55
		DM NDR									37.10	3.72
		Healthy controls									41.57	4.32
		DM NPDR						FAZ area	SCP	Fovea	0.327	0.107
		DM NDR									0.279	0.102
		Healthy controls									0.207	0.037
Veiby et al.^68^	Nidek Co, RS-3000 AOCT	DM severe NPDR	27.6	Cross-sec	483 (254)	109/145	3 × 3	VD	SCP	Fovea	18.15	0.34
		DM moderate NPDR	27.1								16.94	2.22
		DM mild NPDR	25.3								17.02	2.86
		DM NDR	23.5								16.57	3.53
		Healthy controls	23.9								17.98	3.52
		DM severe NPDR						VD	DCP	Fovea	27.89	2.79
		DM moderate NPDR									33.23	2.91
		DM mild NPDR									35.53	1.92
		DM NDR									36.60	2.49
		Healthy controls									38.55	1.83
		DM severe NPDR						FAZ area	SCP	Fovea	0.77	0.58
		DM moderate NPDR									0.29	0.15
		DM mild NPDR									0.28	0.12
		DM NDR									0.25	0.10
		Healthy controls									0.26	0.09
		DM severe NPDR						FAZ area	DCP	Fovea	0.83	0.55
		DM moderate NPDR									0.39	0.16
		DM mild NPDR									0.34	0.12
		DM NDR									0.33	0.11
		Healthy controls									0.35	0.09
Zeng et al.^69^	Optovue, Avanti	DM severe NPDR	56.5	Cross-sec	170 (170)	89/81	6 × 6	VD	SCP	Para-foveal	44.57	4.88
		DM moderate NPDR	57.9								48.42	4.58
		DM mild NPDR	56.9								49.61	5.07
		DM NDR	59.6								50.50	4.11
		Healthy controls	56.1								52.79	3.29
		DM severe NPDR						VD	DCP	Para-foveal	48.15	4.42
		DM moderate NPDR									49.74	4.25
		DM mild NPDR									52.64	3.72
		DM NDR									52.72	4.62
		Healthy controls									55.62	4.60
Li Rudvan et al.^70^	Optovue, Avanti	Pre DM	Unknown	Cross-sec	89 (89)	45/54	3 × 3	VD	SCP	Whole	54.20	3.08
		Healthy controls									54.29	2.89
		Pre DM						VD	SCP	Para-foveal	56.48	3.53
		Healthy controls									56.68	3.18
		Pre DM						VD	SCP	Fovea	28.71	6.08
		Healthy controls									29.78	5.17
		Pre DM						VD	DCP	Whole	60.46	2.14
		Healthy controls									60.93	2.76
		Pre DM						VD	DCP	Para-foveal	63.47	2.77
		Healthy controls									63.71	2.70
		Pre DM						VD	DCP	Fovea	28.77	7.26
		Healthy controls									29.04	6.67
Niestrata-Ortiz et al.^71^	Topcon, Triton	DM > 10 years	16.0	Cross-sec	142 (142)	81/61	3 × 3	FAZ area	SCP	Fovea	0.308	0.14
		DM 5–10 years	13.6								0.293	0.12
		DM < 5 years	12.3								0.315	0.116
		Healthy controls	11.8								0.286	0.127
		DM > 10 years						FAZ area	DCP	Fovea	0.544	0.19
		DM 5–10 years									0.524	0.16
		DM < 5 years									0.503	0.14
		Healthy controls									0.41	0.12
Oliverio et al.^72^	Topcon, Triton	T1DM NDR	34.1	Cross-sec	300 (268)	169/131	3 × 3	VD	SCP	Fovea	21.10	3.60
		T2DM NDR	61.5								21.80	4.10
		Healthy controls	49.5								22.60	5.10
		T1DM NDR						VD	DCP	Fovea	37.20	5.90
		T2DM NDR									37.50	6.10
		Healthy controls									38.10	6.10
		T1DM NDR						FAZ area	SCP	Fovea	0.3	0.80
		T2DM NDR									0.28	0.90
		Healthy controls									0.27	0.10
		T1DM NDR						FAZ area	DCP	Fovea	0.34	0.90
		T2DM NDR									0.32	0.10
		Healthy controls									0.31	0.10
Stulova et al.^73^	Topcon, Triton	T1DM NDR	26	Case–control	131(72)	26/46	3 × 3	VD	SVP	Para-foveal	28.00	2.26
		Healthy controls	25								28.79	1.99
		T1DM NDR						VD	DCP	Para-foveal	17.33	1.39
		Healthy controls									18.14	2.01
		T1DM NDR						FAZ area	SVP	Fovea	0.272	0.095
		Healthy controls									0.254	0.076
Niestrata-Ortiz et al.^74^	Topcon, Triton	T1DM Male	13.86	Cross-sec	142 (142)	81/61	3 × 3	FAZ area	SCP	Fovea	0.266	0.180
		T1DM Female	13.68								0.342	0.118
		Healthy controls Male	12.27								0.261	0.100
		Healthy controls Female	10.53								0.348	0.261
		T1DM Male					3 × 3	FAZ area	SCP	Fovea	0.474	0.138
		T1DM Female									0.572	0.167
		Healthy controls Male									0.281	0.111
		Healthy controls Female									0.572	0.167
Toto et al.^75^	Optovue, Avanti	DME	62.3	Cross-sec	50 (50)	24/26	3 × 3	VD	SCP	Whole	40.70	4.50
		Healthy controls	61.8								50.20	3.60
		DME						VD	SCP	Para-foveal	41.30	4.80
		Healthy controls									51.70	4.30
		DME						VD	SCP	Fovea	29.60	5.40
		Healthy controls									32.80	7.80
		DME						VD	DCP	Whole	45.10	5.20
		Healthy controls									58.50	3.40
		DME						VD	DCP	Para-foveal	47.90	5.10
		Healthy controls									61.10	4.30
		DME						VD	DCP	Fovea	18.90	9.20
		Healthy controls									28.50	8.30
Liu et al.^76^	Optovue, Avanti	Pregnant + GDM	30.6	Cross-sec	179 (99)	0/99	3 × 3	VD	SCP	Whole	48.20	2.60
		Pregnant-GDM	30.7								48.50	2.40
		Healthy controls	30.6								50.40	1.50
		Pregnant + GDM						VD	DCP	Whole	53.30	3.10
		Pregnant-GDM									53.90	2.60
		Healthy controls									50.60	3.50
		Pregnant + GDM						VD	SCP	Para-foveal	51.50	2.60
		Pregnant-GDM									51.80	2.80
		Healthy controls									53.20	1.60
		Pregnant + GDM						VD	DCP	Para-foveal	56.20	2.70
		Pregnant-GDM									57.10	2.70
		Healthy controls									53.00	3.60
		Pregnant + GDM						VD	SCP	Fovea	16.50	6.10
		Pregnant-GDM									14.50	4.30
		Healthy controls									24.00	6.30
		Pregnant + GDM						VD	DCP	Fovea	30.30	7.50
		Pregnant-GDM									27.90	6.80
		Healthy controls									32.20	6.90
		Pregnant + GDM						FAZ area	SCP	Fovea	0.35	0.12
		Pregnant-GDM									0.39	0.1
		Healthy controls									0.31	0.11
Sugimoto et al.^77^	Nidek Co, RS-3000 AOCT	Pregnant + GDM	34.0	Cross-sec	51 (51)	0/51	3 × 3	FAZ area	SCP	Fovea	0.41	0.16
		DM NDR	34.0								0.43	0.1
		Healthy controls	29.6								0.38	0.11
		Pregnant + GDM						FAZ area	DCP	Fovea	0.69	0.16
		DM NDR									0.79	0.25
		Healthy controls									0.78	0.23
Tarek et al.^78^	Optovue, Avanti	Non-diabetes phacoemulsification Diabetes phacoemulsification	57.2	Case–control	60 (60)	15/45	6 × 6	VD	SCP	Fovea	9.27	7.37
			54.5								13.37	6.45
Aschauer et al.^79^	Optovue, Avanti	T2DM +/−DR	57	Cohort (baseline data)	117 (59)	38/21	6 × 6	VD	SVC	Para-foveal	51.00	5.77
								VD	DVC	Para-foveal	52.57	4.11
								FAZ area	SVC	Fovea	0.25	0.12
Sun et al.^80^	Topcon, Triton	Diabetes NDR	62.9	Cohort (baseline data)	205(129)	61/68	3 × 3	VD	SCP	Para-foveal	76.29	7.00
								VD	DCP	Para-foveal	33.99	3.57
								FAZ area	SCP	Fovea	0.40	0.13
								FAZ area	DCP	Fovea	1.09	0.43

VD data is given as percentage (%), FAZ data is given as mm^2^. DM, diabetes mellitus; NDR, no diabetic retinopathy; VD, vessel density; FAZ, foveal avascular zone; cross-sec, cross-sectional; SRL, superficial retinal layer; HD-OCT, high-definition optical coherence tomography; NPDR, non-proliferative diabetic retinopathy; DRL, deep retinal layer; T1DM, Type 1 diabetes mellitus; T2DM, Type 2 diabetes mellitus; PDR, proliferative diabetic retinopathy; SRVP, superficial retinal vascular plexus; DRVP, deep retinal vascular plexus; DME, diabetic macular oedema; GDM, gestational diabetes mellitus.

**Table 2:** Study Characteristics of studies included looking at patients with diseases other than diabetes.

Author, ref	Manufacturer	Diagnosis included	Mean age (years)	Study Design	Number of eyes (number of patients)	Number of males/females	Scan size (mm)	Metric	Vessel layer	Region	Mean	SD
Bulut et al.^81^	Not specified	Late AD	74.2	Cross-sec	52 (52)	24/28	6 × 6	VD	SVP	Whole	45.50	3.85
		Healthy controls	72.6								48.67	3.29
		Late AD						VD	SVP	Para-foveal	47.96	4.86
		Healthy controls									51.12	4.10
		Late AD						VD	SVP	Fovea	29.04	7.17
		Healthy controls									34.80	6.76
Chua et al.^82^	Cirrus HD-OCT 5000	Late AD	74.9	Cross-sec	90 (90)	44/46	3 × 3	VD	SCP	Para-foveal	14.78	1.14
		MCI	77.9								14.94	1.02
		Healthy controls	76.7								15.66	0.96
		Late AD						VD	DCP	Para-foveal	20.42	1.60
		MCI									20.81	1.65
		Healthy controls									21.54	1.55
		Late AD						FAZ area	SCP	Fovea	0.34	0.14
		MCI									0.35	0.12
		Healthy controls									0.31	0.12
		Late AD						FAZ area	DCP	Fovea	1.13	0.43
		MCI									1.24	0.39
		Healthy controls									1.11	0.47
Haan et al.^83^	Zeiss, Cirrus HD-OCT 5000	Late AD	65.4	Cross-sec	86 (86)	49/37	6 × 6	VD	SCP	Para-foveal	17.30	1.50
		Healthy controls	60.6								17.40	1.20
		Late AD						FAZ area	SCP	Fovea	0.24	0.06
		Healthy controls									0.26	0.08
Lahme et al.^84^	Optovue, Avanti	Early AD	70.0	Cross-sec	74 (74)	29/44	3 × 3	VD	SCP	Whole	48.77	3.92
		Healthy Controls	66.1								51.64	3.28
		Early AD						VD	SCP	Para-foveal	50.93	4.05
		Healthy Controls									53.55	3.31
		Early AD						VD	SCP	Fovea	29.40	5.72
		Healthy Controls									31.06	5.35
		Early AD						VD	DCP	Whole	55.35	3.16
		Healthy Controls									56.72	2.21
		Early AD						VD	DCP	Para-foveal	57.97	3.30
		Healthy Controls									58.38	4.64
		Early AD						VD	DCP	Fovea	31.21	6.60
		Healthy Controls									29.32	6.67
		Early AD						FAZ area	SCP	Fovea	0.28	0.08
		Healthy Controls									0.28	0.09
		Early AD						FAZ area	DCP	Fovea	0.32	0.10
		Healthy Controls									0.33	0.14
Robbins et al.^85^	Zeiss, Cirrus HD-OCT 5000	Early AD	62.4	Cross-sec	224 (122)	44/78	3 × 3	VD	SCP	Whole	20.15	1.97
		Late AD	76.9								18.55	2.45
		Healthy controls	68.1								20.36	1.50
		Early AD						VD	SCP	Para-foveal	21.22	1.90
		Late AD									19.56	2.46
		Healthy controls									21.40	1.47
		Early AD						FAZ area	SCP	Fovea	0.21	0.09
		Late AD									0.25	0.14
		Healthy controls									0.23	0.10
		Early AD					6 × 6	VD	SCP	Whole	17.97	1.09
		Late AD									16.96	2.06
		Healthy controls									17.71	1.13
		Early AD						VD	SCP	Para-foveal	18.10	1.36
		Late AD									16.90	2.55
		Healthy controls									17.72	1.34
Wang X et al.^86^	Optovue, Avanti	Late AD	71.8	Cross-sec	158 (158)	96/62	3 × 3	VD	SCP	Whole	44.66	3.36
		MCI	72.7								44.00	3.07
		Healthy controls	69.5								46.82	2.08
		Late AD						VD	DCP	Whole	49.42	3.40
		MCI									49.57	2.89
		Healthy controls									50.89	2.86
		Late AD						VD	SCP	Para-foveal	47.70	3.76
		MCI									47.12	3.35
		Healthy controls									49.86	2.26
		Late AD						VD	DCP	Para-foveal	52.02	3.65
		MCI									52.36	2.96
		Healthy controls									53.40	2.77
		Late AD						VD	SCP	Fovea	15.89	5.34
		MCI									14.09	5.21
		Healthy controls									16.18	5.27
		Late AD						VD	DCP	Fovea	28.53	6.80
		MCI									26.83	7.11
		Healthy controls									28.94	6.70
Wu et al.^87^	Optovue, Avanti	Late AD	69.9	Cross-sec	88 (60)	33/27	6 × 6	VD	SCP	Para-foveal	49.56	2.81
		MCI	67.8								50.37	2.33
		Healthy controls	68.7								50.47	2.73
		Late AD						VD	DCP	Para-foveal	43.10	2.75
		MCI									48.09	3.88
		Healthy controls									52.28	2.89
		Late AD						FAZ area	Full	Fovea	0.44	0.08
		MCI									0.37	0.06
		Healthy controls									0.26	0.07
Zabel et al.^88^	Optovue, Avanti	Late AD	74.1	Cross-sec	81 (81)	24/47	6 × 6	VD	SVP	Whole	47.92	3.04
		POAG	71.9								39.72	4.97
		Healthy controls	74.3								48.15	3.03
		Late AD						VD	DVP	Whole	43.95	5.15
		POAG									47.44	6.07
		Healthy controls									49.46	4.27
Zabel et al.^89^	Optovue, Avanti	Late AD	74.4	Cross-sec	168 (108)	41/68	6 × 6	VD	SVP	Whole	46.80	3.20
		POAG	72.1								42.40	5.40
		Healthy controls	71.4								48.50	3.40
		Late AD						VD	DVP	Whole	45.00	4.70
		POAG									47.60	5.20
		Healthy controls									48.50	5.10
		Late AD						VD	SVP	Para-foveal	49.40	4.00
		POAG									46.70	5.50
		Healthy controls									51.40	4.30
		Late AD						VD	DVP	Para-foveal	51.70	3.60
		POAG									53.50	4.10
		Healthy controls									53.20	3.40
		Late AD						VD	SVP	Fovea	19.70	6.20
		POAG									18.40	5.70
		Healthy controls									23.90	6.60
		Late AD						VD	DVP	Fovea	34.30	7.30
		POAG									34.70	7.60
		Healthy controls									39.60	5.60
Yan et al. 2021 ^90^	Optovue, Avanti	Mild AD	Un-known	Cross-sec	116(63)	Unknown	3 × 3	VD	SCP	Fovea	15.80	6.92
		Healthy controls									15.94	6.26
		Mild AD						VD	DCP	Fovea	28.80	8.15
		Healthy controls									28.90	8.30
		Mild AD						VD	SCP	Para-foveal	46.62	5.14
		Healthy controls									48.61	3.79
		Mild AD						VD	DCP	Para-foveal	51.57	3.68
		Healthy controls									52.63	3.86
Shin et al.^91^	Zeiss, Cirrus HD-OCT 5000	MCI	72.8	Case control	77 (55)	42/13	6 × 6	VD	SCP	Para-foveal	14.00	3.90
		Healthy controls	69.0								25.50	1.90
		MCI						VD	DCP	Para-foveal	16.30	2.50
		Healthy controls									25.60	1.80
		MCI						FAZ area	SCP	Fovea	0.31	0.11
		Healthy controls									0.27	0.09
		MCI						FAZ area	DCP	Fovea	0.95	0.24
		Healthy controls									0.80	0.20
Rascuna et al.^92^	Optovue, Avanti	PD	61.5	Cross-sec	111 (57)	32/25	3 × 3	VD	SCP	Whole	44.60	4.40
		iRBD	58.8								43.00	4.60
		Healthy controls	65.1								43.90	3.80
		PD						VD	DCP	Whole	47.80	4.30
		iRBD									50.50	3.10
		Healthy controls									46.10	4.30
		PD						VD	SCP	Para-foveal	46.90	4.50
		iRBD									45.70	5.10
		Healthy controls									46.10	4.30
		PD						VD	DCP	Para-foveal	49.80	4.50
		iRBD									52.50	3.40
		Healthy controls									49.90	3.60
		PD						VD	SCP	Fovea	19.30	5.70
		iRBD									15.60	4.80
		Healthy controls									18.40	5.90
		PD						VD	DCP	Fovea	33.80	6.60
		iRBD									31.60	5.80
		Healthy controls									32.90	7.90
Robbins et al.^93^	Zeiss, Cirrus HD-OCT 5000	PD	71.7	Cross-sec	372 (206)	116/90	6 × 6	VD	SCP	Whole	17.34	1.38
		Healthy controls	70.9								17.69	1.46
		PD						VD	SCP	Para-foveal	17.17	1.74
		Healthy controls									17.75	1.68
		PD						FAZ area	SCP	Fovea	0.22	0.10
		Healthy controls									0.23	0.11
Zou et al.^94^	Zeiss, Angioplex	PD	61.9	Cross-sec	70 (70)	36/34	6 × 6	FAZ area	SCP	Fovea	0.31	0.12
		Healthy controls	60.2								0.29	0.10
Liu B et al.^95^	Optovue, Avanti	Stroke	62.0	Cross-sec	384 (384)	210/174	6 × 6	VD	SCP	Whole	47.45	4.35
		Healthy controls	61.7								49.44	3.71
		Stroke						VD	DCP	Whole	47.64	6.07
		Healthy controls									50.75	6.29
		Stroke						VD	SCP	Para-foveal	49.23	5.56
		Healthy controls									51.78	4.67
		Stroke						VD	DCP	Para-foveal	52.26	5.10
		Healthy controls									55.17	4.70
		Stroke						VD	SCP	Fovea	17.73	6.72
		Healthy controls									18.55	7.26
		Stroke						VD	DCP	Fovea	32.72	7.36
		Healthy controls									32.67	7.45
Aly et al.^96^	Optovue, Avanti	NMOSD-ON	46.6	Cross-sec	114/58	13/45	6 × 6	VD	SVC	Para-foveal	51.00	3.80
		NMOSD + ON	46.6								47.40	4.30
		MS-ON	38.0								51.80	2.60
		MS + ON	38.0								50.40	3.70
		Healthy controls	42.0								53.30	2.50
		NMOSD-ON						VD	DVC	Para-foveal	56.90	5.10
		NMOSD + ON									57.00	3.90
		MS-ON									57.20	5.70
		MS + ON									59.10	3.90
		Healthy controls									57.30	5.50
		NMOSD-ON						FAZ area	SVC	Fovea	0.29	0.09
		NMOSD + ON									0.32	0.09
		MS-ON									0.22	0.10
		MS + ON									0.28	0.14
		Healthy controls									0.20	0.07
Cordon et al.^97^	Topcon, Triton	MS-ON	41.7	Cross-sec	241 (241)	32/209	6 × 6	VD	SVP	Para-foveal	21.45	4.51
		Healthy controls	41.8								21.89	4.80
Karaküçük et al.^98^	Topcon, Triton	MS-ON	36.5	Cross-sec	130 (130)	91/39	6 × 6	FAZ area	SCP	Fovea	0.15	0.05
		Healthy controls	35.3								0.16	0.07
		MS-ON						FAZ area	DCP	Fovea	0.23	0.05
		Healthy controls									0.24	0.13
Yilmaz et al.^99^	Nidek Co, RS-3000 AOCT	MS + ON	39.3	Cross-sec	216 (108)	20/88	4.5 × 4.5	VD	SCP	Whole	38.05	4.97
		MS-ON	39.3								41.25	4.42
		Healthy controls	38.6								42.35	2.68
		MS + ON						VD	DCP	Whole	32.11	7.81
		MS-ON									34.69	5.96
		Healthy controls									38.21	4.53
		MS + ON						FAZ area	SCP	Fovea	0.34	0.11
		MS-ON									0.33	0.13
		Healthy controls									0.30	0.09
Criscuolo et al.^100^	Optovue, Avanti	aMCI	73.0	Cross-sec	112 (56)	26/30	6 × 6	VD	SCP	Whole	44.92	5.04
		Healthy controls	73.1								48.12	4.53
		aMCI						VD	DCP	Whole	45.13	6.67
		Healthy controls									50.58	4.69
		aMCI						FAZ Area	Full	Fovea	0.28	0.12
		Healthy controls									0.19	0.06
Zhang Y et al.^101^	Optovue, Avanti	Large artery atherosclerosis	60.1	Cross-sec	180 (180)	134/46	6 × 6	VD	SCP	Whole	45.59	4.26
		Small vessel occlusion	58.8								46.72	3.13
		Healthy controls	59.0								45.65	2.82
		Large artery atherosclerosis						VD	DCP	Whole	47.49	3.12
		Small vessel occlusion									48.11	3.70
		Healthy controls									49.46	3.14
Wang et al.^102^	Optovue, Avanti	CSVD	63.9	Cross-sec	152 (77)	41/36	3 × 3	FAZ	DCP	Fovea	0.33	0.13
		Healthy controls	61.3								0.34	0.14
Zhang et al.^103^	Zeiss, Cirrus HD-OCT 5000	Cerebrovascular disease	56.0	Cross-sec	295 (165)	118/47	6 × 6	VD	SCP	Para-foveal	16.21	2.11
		Healthy controls	53.2								18.19	1.07
		Cerebrovascular disease						VD	SCP	Fovea	6.93	2.96
		Healthy controls									8.81	2.84
		Cerebrovascular disease						FAZ area	SCP	Fovea	0.306	0.12
		Healthy controls									0.306	0.11
Kazanci et al.^104^	Optovue, Avanti	β-thalassemia	13.6	Cross-sec	62 (62)	30/32	6 × 6	VD	SCP	Whole	51.58	2.01
		Healthy controls	12.6								51.90	2.08
		β-thalassemia						VD	DCP	Whole	53.44	5.80
		Healthy controls									55.54	5.58
		β-thalassemia						VD	SCP	Fovea	21.67	6.65
		Healthy controls									22.90	6.11
		β-thalassemia						VD	DCP	Fovea	39.55	7.95
		Healthy controls									38.98	8.54
		β-thalassemia						VD	SCP	Para-foveal	54.05	2.61
		Healthy controls									54.40	3.76
		β-thalassemia						VD	DCP	Para-foveal	56.91	4.81
		Healthy controls									58.62	4.56
		β-thalassemia						FAZ area	SCP	Fovea	0.265	0.11
		Healthy controls									0.296	0.12
Peng et al.^105^	Optovue, Avanti	CKD	62.4	Case control	326 (326)	184/142	3 × 3	VD	SVP	Para-foveal	46.90	4.50
		Healthy controls	63.0								49.00	3.70
		CKD						VD	DVP	Para-foveal	50.90	3.90
		Healthy controls									52.00	3.10
Wang et al.^106^	Topcon, Triton	DM moderate-severe CKD	72.6	Cross-sec	874 (874)	353/521	3 × 3	VD	SCP	Whole	44.50	1.30
		DM mild CKD	65.0								45.3	1.8
		DM no CKD	60.4								45.7	1.5
		DM moderate-severe CKD						VD	SCP	Para-foveal	47.2	1.7
		DM mild CKD									48.4	1.9
		DM no CKD									49.1	2.1
		DM moderate-severe CKD						VD	SCP	Fovea	20.4	5.3
		DM mild CKD									19.3	5.2
		DM no CKD									20.1	5.0
Cankurtaran et al.^107^	Optovue, Avanti	Diabetes normo-albuminuria	55.7	Cross-sec	137 (137)	69/68	6 × 6	VD	SCP	Whole	49.70	2.71
		Diabetes microalbuminuria	56.7								47.27	3.99
		Healthy controls	54.8								50.43	2.61
		Diabetes normo-albuminuria						VD	DCP	Whole	50.43	5.76
		Diabetes microalbuminuria									49.08	7.06
		Healthy controls									53.59	6.04
		Diabetes normo-albuminuria						VD	SCP	Para-foveal	52.25	3.64
		Diabetes microalbuminuria									49.88	4.87
		Healthy controls									53.44	3.57
		Diabetes normo-albuminuria						VD	DCP	Para-foveal	55.30	4.19
		Diabetes microalbuminuria									53.61	5.04
		Healthy controls									55.97	4.61
		Diabetes normo-albuminuria						VD	SCP	Fovea	18.52	5.08
		Diabetes microalbuminuria									17.94	6.04
		Healthy controls									2.13	5.81
		Diabetes normo-albuminuria						VD	DCP	Fovea	34.29	5.89
		Diabetes microalbuminuria									33.94	8.61
		Healthy controls									37.52	6.85
Değirmenci et al.^108^	Optovue, Avanti	Behcet’s-ocular involvement	45.7	Cross-sec	23 (12)	27/15	6 × 6	FAZ area	SCP	Fovea	0.331	0.121
		Healthy controls	51.4		49 (29)						0.240	0.072
		Behcet’s-ocular involvement						FAZ area	DCP	Fovea	0.352	0.126
		Healthy controls									0.257	0.070
Smid et al.^109^	Heidelberg, Spectralis	Behcet’s + ocular involvement	51.0	Cross-sec	68 (68)	38/20	3 × 3	VD	SCP	Para-foveal	30.0	9.00
		Behcet’s-ocular involvement	48.0								36.00	4.00
		Healthy controls	44.0								38.90	1.60
		Behcet’s + ocular involvement						VD	DCP	Para-foveal	25.00	7.00
		Behcet’s-ocular involvement									30.00	4.00
		Healthy controls									33.50	1.90
Yilmaz et al.^110^	Optovue, Avanti	Behcet’s + ocular involvement	36.0	Cross-sec	70 (70)	26/44	6 × 6	VD	SCP	Para-foveal	41.70	6.90
		Behcet’s-ocular involvement	40.1								47.30	4.40
		Healthy controls	39.6								47.90	7.20
		Behcet’s + ocular involvement						VD	SCP	Fovea	20.10	7.30
		Behcet’s-ocular involvement									18.90	9.90
		Healthy controls									19.50	9.40
		Behcet’s + ocular involvement						VD	DCP	Para-foveal	47.20	6.30
		Behcet’s-ocular involvement									52.70	3.70
		Healthy controls									52.90	4.20
		Behcet’s + ocular involvement						VD	DCP	Fovea	32.80	8.90
		Behcet’s-ocular involvement									34.50	10.00
		Healthy controls									32.11	7.81
Aksoy et al.^111^	Optovue, Avanti	Uveitis	38.0	Cross-sec	65 (65)	33/32	6 × 6	VD	SCP	Para-foveal	49.06	5.56
		Healthy controls	37.0								55.85	2.93
		Uveitis						VD	SCP	Fovea	32.57	5.43
		Healthy controls									32.59	4.07
		Uveitis						VD	DCP	Para-foveal	55.60	7.22
		Healthy controls									66.02	1.79
		Uveitis						VD	DCP	Fovea	34.06	4.49
		Healthy controls									34.91	7.81
Agarwal et al.^112^	Topcon, Triton	Uveitis	34.7	Cross-sec	68 (50)	29/21	3 × 3	FAZ area	SCP	Fovea	0.34	0.08
		Healthy controls	33.6								0.26	0.08
Kim et al.^6^	Zeiss, Prototype	Uveitis	Unknown	Cross-sec	155 (92)	37/55	3 × 3	VD	SRL	Para-foveal	37.80	4.10
		Healthy controls	Unknown								42.60	1.90
		Uveitis						VD	DRL	Para-foveal	41.20	2.90
		Healthy controls									42.50	1.70
Tian et al.^113^	Zeiss, PLEX Elite 9000	Uveitis + vasculitis	45.9	Cross-sec	92 (58)	26/32	3 × 3	FAZ area	SCP	Fovea	0.20	0.10
		Uveitis-vasculitis	45.9								0.10	0.11
		Healthy controls	42.0								0.30	0.50
Fan et al.^114^	Optovue, Avanti	VKHD + SGF	40.6	Cross-sec	106 (53)	23/30	3 × 3	VD	SCP	Whole	44.8	2.40
		VKHD-SGF	38.0								47.00	2.30
		Healthy controls	39.6								47.70	1.90
		VKHD + SGF						VD	SCP	Para-foveal	47.70	2.70
		VKHD-SGF									50.20	2.20
		Healthy controls									50.70	2.00
		VKHD + SGF						VD	SCP	Fovea	14.50	8.80
		VKHD-SGF									16.50	6.70
		Healthy controls									18.80	4.20
		VKHD + SGF						VD	DCP	Whole	47.70	2.50
		VKHD-SGF									51.40	2.60
		Healthy controls									51.60	2.80
		VKHD + SGF						VD	DCP	Para-foveal	50.50	2.70
		VKHD-SGF									53.90	2.70
		Healthy controls									53.80	3.20
		VKHD + SGF						VD	DCP	Fovea	26.70	11.40
		VKHD-SGF									30.60	7.60
		Healthy controls									33.50	4.60
Karaca et al.^115^	Optovue, Avanti	Inactive VKHD	39.9	Cross-sec	51 (51)	23/28	6 × 6	VD	SCP	Whole	50.60	4.70
		Healthy controls	38.9								54.30	2.60
		Inactive VKHD						VD	SCP	Para-foveal	53.50	4.80
		Healthy controls									56.70	2.80
		Inactive VKHD						VD	SCP	Fovea	18.20	6.90
		Healthy controls									24.60	3.40
		Inactive VKHD						VD	DCP	Whole	53.10	4.60
		Healthy controls									61.10	2.80
		Inactive VKHD						VD	DCP	Para-foveal	55.90	3.40
		Healthy controls									61.90	3.10
		Inactive VKHD						VD	DCP	Fovea	33.60	6.90
		Healthy controls									41.90	3.80
Aksoy et al.^116^	Optovue, Avanti	Fuch’s eye	34.3	Cross-sec	30 (30)	14/16	6 × 6	VD	SCP	Fovea	18.69	6.91
		Fellow eye (no Fuch’s)	34.3		30 (30)	14/16					30.23	6.90
		Healthy control	35.5		30 (30)	14/16					31.58	4.07
		Fuch’s eye						VD	DCP	Fovea	33.83	6.18
		Fellow eye (no Fuch’s)									39.63	6.01
		Healthy control									34.06	4.49
		Fuch’s eye						VD	SCP	Para-foveal	45.56	6.56
		Fellow eye (no Fuch’s)									52.28	6.26
		Healthy control									55.85	2.93
		Fuch’s eye						VD	DCP	Para-foveal	54.01	5.15
		Fellow eye (no Fuch’s)									64.11	4.83
		Healthy control									65.02	4.75
		Fuch’s eye						FAZ area	SCP	Fovea	0.39	0.25
		Fellow eye (no Fuch’s)									0.36	0.25
		Healthy control									0.30	0.25

VD data is given as percentage (%), FAZ data is given as mm^2^. AD, Alzheimer's disease; cross-sec, cross-sectional; VD, vessel density; MCI, mild cognitive impairment; FAZ, foveal avascular zone; POAG, primary open angle glaucoma; PD, Parkinson’s disease; iRBD, idiopathic rapid-eye-movement sleep behaviour disorder; NMOSD-ON, neuromyelitis optica spectra disorder without optic neuritis; NMOSD + ON, neuromyelitis optica spectra disorder with optic neuritis; MS-ON, multiple sclerosis without optic neuritis; MS + ON, multiple sclerosis with optic neuritis; aMCI, amnestic mild cognitive impairment; CSVD, cerebral small vessel disease; CKD, chronic kidney disorder; Behcet's-ocular involvement, Behcet's without ocular involvement; Behcet's + ocular invlvement, Behcet's with ocular involvement; VKHD + SGF, Vogt-Koyanagi-Harada disease with sunset glow fundus; VKHD-SGF, Vogt-Koyanagi-Harada disease without sunset glow fundus; VKHD, Vogt-Koyanagi-Harada disease.

Papers from Hirano^117^, Karst^118^, Marques^119^, Vujosevic^120^ and Yoon^121^ were not presented as they did not include VD or FAZ data.

### Heterogeneity of assessments

The included studies recruited patients with 64 different diagnoses, used seven different OCTA systems (Table 3), defined six different volume scan densities, with four different volume scan sizes, nine different perfusion analysis methods, five different vessel layer definitions for superficial and deep capillary plexi, and examined three different macula regions, giving a total of 197 distinct methods of assessing retinal perfusion, but a potential of more than 7000 different combinations (Table 4). Heterogeneity in OCTA analysis limited data synthesis, however the most studied condition was diabetes mellitus with or without diabetic retinopathy and the most reported analysis methods were VD and FAZ area. We therefore present detailed synthesis of VD and FAZ area in diabetes mellitus.

**Table 3:** OCTA equipment and software in included studies.

Company	Instrument	Source	Software	µm between B-scan
Zeiss	Cirrus HD-OCT 5000	SD-OCT	OMAG	12.2
Zeiss	PLEX Elite 9000	SS-OCT	OMAG	30
Zeiss	AngioPlex	SD-OCT	OMAG	Unknown
Optovue	Avanti	SD-OCT	SS-ADA	9.9
Topcon	Triton	SS-OCT	OCTARA	9.4
Nidek Co	RS-3000 AOCT	SD-OCT	CODAA	11.7
Heidelberg	OCT2	SD-OCT	FSPA	5.7

Kim et al.[6] used a Zeiss prototype instrument that is not included in this table. OCTA, optical coherence tomography angiography; SD-OCT, Spectral-domain optical coherence tomography; SS-OCT, swept-source optical coherence tomography; OMAG, optical microangiography; SS-ADA, split-spectrum amplitude decorrelation angiography; OCTARA, optical coherence tomography angiography ratio analysis; CODAA, complex optical coherence tomography signal difference analysis angiography; FSPA, full spectrum probabilistic approach.

**Table 4:** Different parameters available for assessing retinal perfusion in included studies.

Macula volume dimension	A-scans in volume	Macula region of interest	Perfusion metric	Retinal layer	Retinal layer definition
3 × 3 mm	245 × 245	Foveal	VD	SCP	NFL + GCL + IPL^122^
6 × 6 mm	256 × 256	Parafoveal	SVD	DCP	INL + OPL^122^
12 × 12 mm	304 × 304	Whole macula (9 or 36 mm^2^)	VLD	SVP	GCL + IPL^122^
4.5 × 4.5 mm	320 × 320		FD	DVP	IPL + INL + OPL ^105^
	400 × 400		SFD	SVC	NFL + GCL + IPL^122^
	512 × 512		PD	DVC	IPL + INL + OPL^122^
			FAZ area	SRL	Inner 60–70% of the whole retina (ILM-RPE) ^6,18^ or NFL + GCL + IPL ^52^
			FAZ perimeter	DRL	Outer 30–40% of the inner retina (ILM-RPE)^6,18^ or IPL + INL + OPL^52,117^
			FAZ acircularity ratio	SCC	NFL + GCL + IPL ^118^
			FAZ acircularity index	DCC	IPL + INL + OPL ^118^

VD, vessel density; SVD, skeletonised vessel density; VLD, vessel length density; FD, fractal dimension; SFD, skeletonised fractal dimension; PD, perfusion density; FAZ, foveal avascular zone; NFL, nerve fibre layer; GCL, ganglion cell layer; IPL, inner plexiform layer; INL, inner nuclear layer; OPL, outer plexiform layer; SCP, superficial capillary plexus; DCP, deep capillary plexus; SVP, superficial vascular plexus; DVP, deep vascular plexus; SVC, superficial vascular plexus; DVC, deep vascular plexus; SRL, superficial retinal layer; DRL, deep retinal layer; SCC, superficial capillary complex; DCC, deep capillary complex. Retinal layer illustrations are in Supplementary Fig. 1.

### Risk of bias results

A risk of bias analysis using the NIH quality assessment tool for observational cohort and cross-sectional studies (14 questions) and the NIH tool of case–control studies (12 questions) graded 23 studies as good, 47 as fair, and 18 as poor quality (Supplementary Table 3). We retained studies rated as poor quality to illustrate heterogeneity.

**Figure 2:**
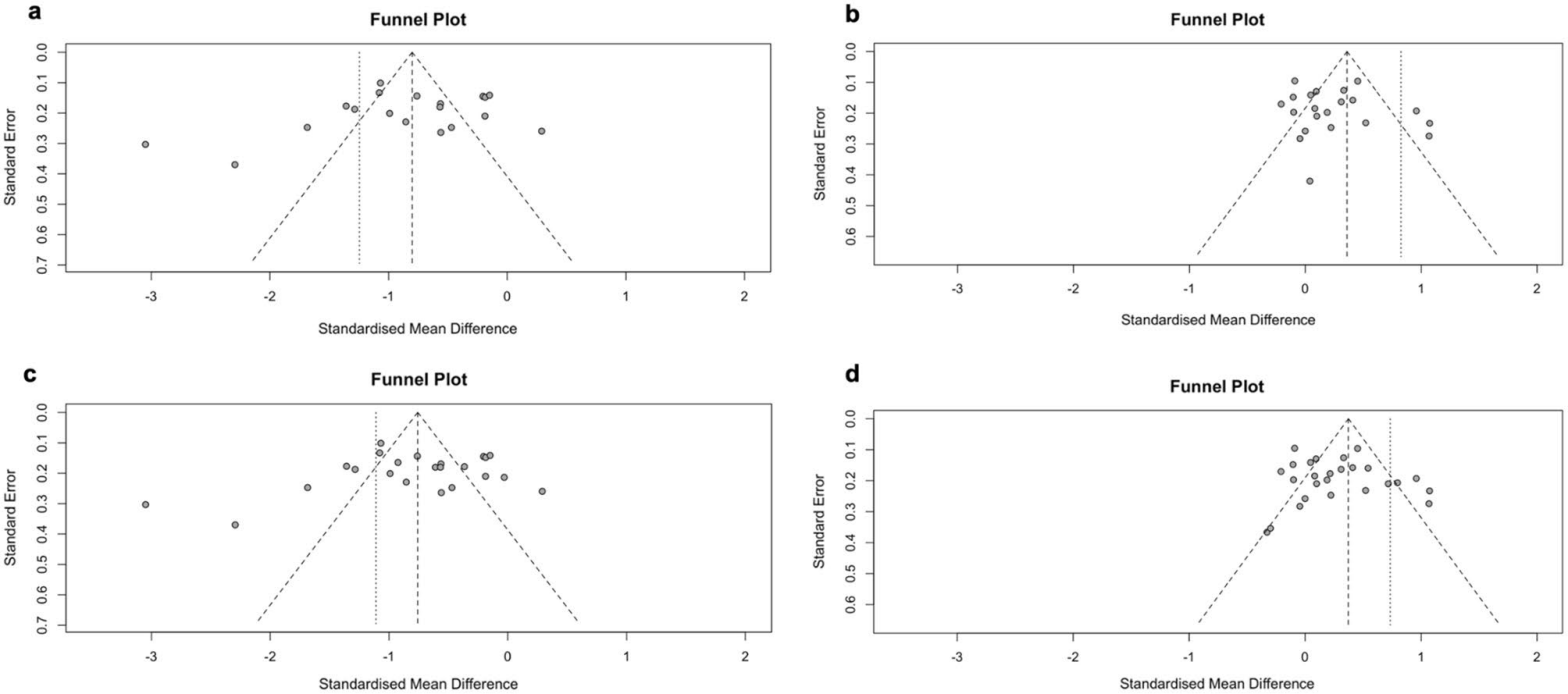
Funnel plots of the effect of diabetic retinopathy on retinal perfusion. (**a**) VD in patients with diabetic eye disease. (**b**) FAZ in patients with diabetic eye disease. (**c**) VD in all patients with diabetes mellitus. (**d**) FAZ in all patients with diabetes mellitus. VD, vessel density; FAZ, foveal avascular zone.

Bias was identified predominantly in question 6 (“were the exposure(s) of interest measured prior to the outcome(s)?”), question 7 (“Was the timeframe sufficient so that one could reasonably expect to see an association?”) and question 10 (“Was the exposure(s) assessed more than once?”) of the NIH quality assessment tools because the included studies were mostly cross-sectional and not longitudinal by design. Sample size justification was rarely given (question 5) and study population was not always explicitly defined (question 2). Funnel plots (Fig. 2) showed no evidence of publication bias.

### Effect of non-proliferative diabetic retinopathy on retinal perfusion

**Figure 3:**
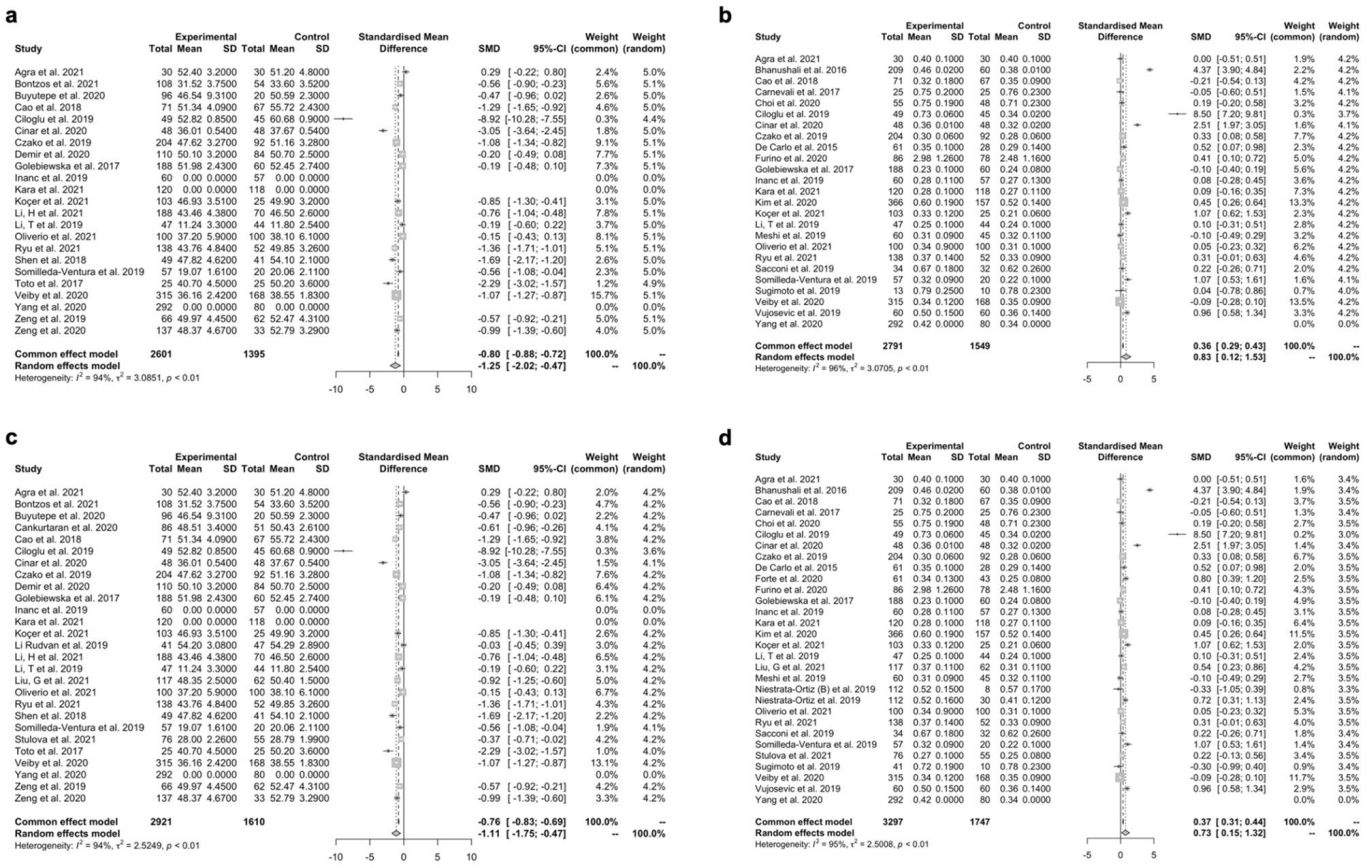
Meta-analysis forest plots for diabetic retinopathy. Forest plots analysing the effect of diabetic retinopathy on retinal perfusion by comparing healthy controls with diabetic retinopathy patients for (**a**) VD%, and (**b**) FAZ and; comparing healthy controls with all diabetic patients for (**c**) VD% and (**d**) FAZ comparing healthy controls with all diabetic patients with diabetic retinopathy and without diabetic retinopathy. VD%, percentage vessel density; FAZ, foveal avascular zone; SD, standard deviation.

Twenty-six papers were included, as they had VD% results calculated from the same vessel layer, vascular region, and used the same scan size. In comparison to healthy controls, eyes with diabetic eye disease had, on average, a smaller VD% of − 3.52% (n = 18 studies, 95% CI [− 6.71; − 0.32], *p* = 0.031; Fig. 3b) and a larger FAZ area of 1.50 mm^2^ (n = 26 studies, 95% CI [0.2999; 2.7007], *p* = 0.014; Fig. 3a). In comparison to healthy controls, eyes of patients with diabetes had, on average, a smaller VD% of − 1.7822% (95% CI [− 3.4935; − 0.0708], *p* = 0.041; Fig. 3d) and a larger FAZ area (0.7046 mm^2^ (95% CI [0.1826; 1.2266], *p* = 0.0082; Fig. 3c). Study characteristics are summarised in Table 1.

### Effect of analysis methods on the detection of diabetes without diabetic retinopathy

**Figure 4:**
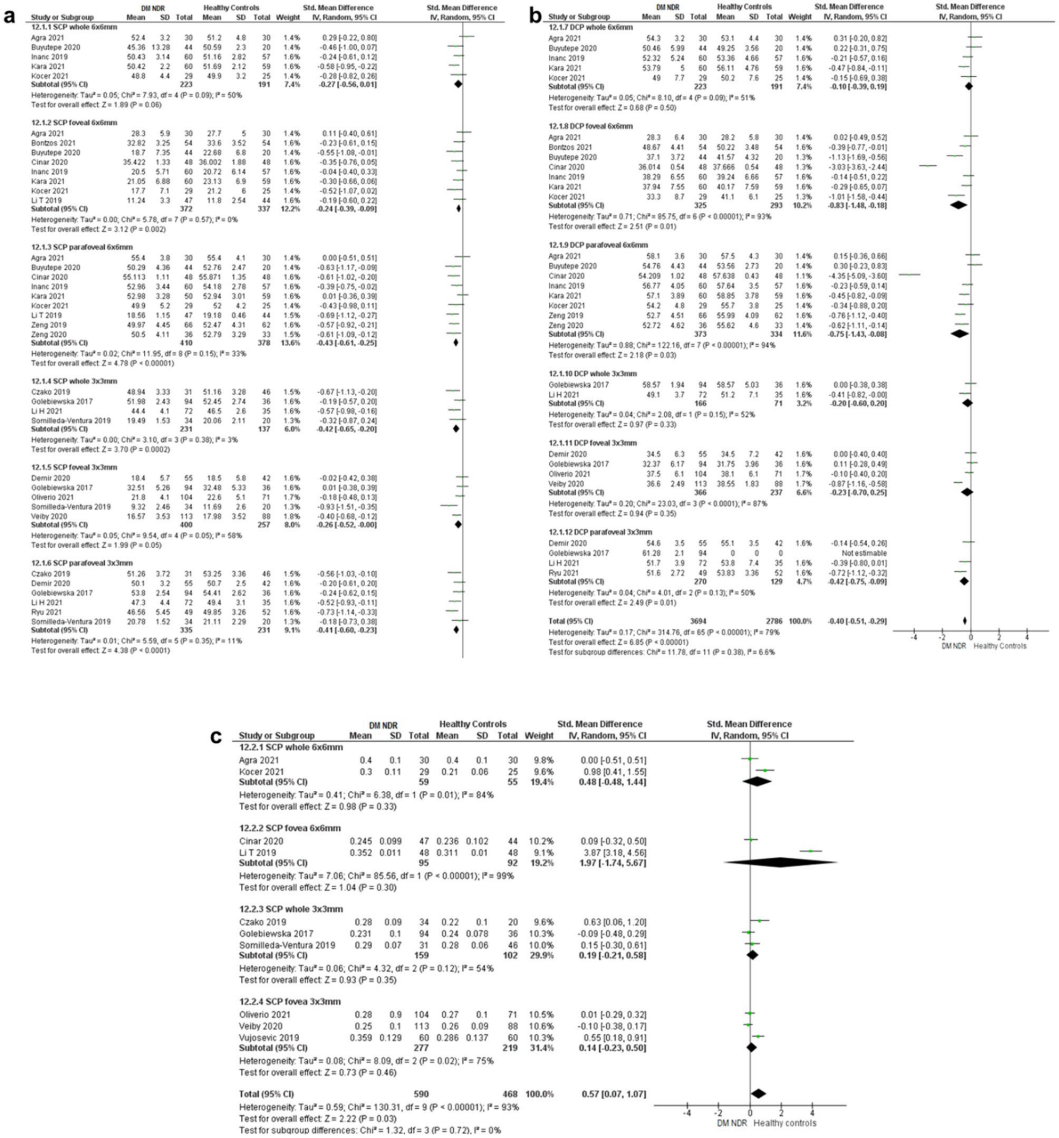
Meta-analysis forest plots for diabetes without diabetic retinopathy. Forest plots showing the effect of analysis methods on the detection of altered retinal perfusion in diabetes without diabetic retinopathy versus healthy controls measured by VD% and FAZ area, grouping studies depending on scan size, vessel layer, and macular region used to derive results. (**a**,**b**) VD%. (**c**) FAZ area. VD%, percentage vessel density; FAZ, foveal avascular zone; DM NDR, diabetes mellitus without diabetic retinopathy; SD, standard deviation; SCP, superficial capillary plexus; DCP, deep capillary plexus.

Nineteen papers were included. All included analysis methods across the different vascular plexi and retinal areas detected reduced perfusion in patients with diabetes without diabetic retinopathy compared to healthy controls (Fig. 4), although even within individual analysis methods, such as in the deep capillary plexus (DCP) with foveal perfusion assessed from a 6 × 6 mm macular volume, heterogeneity was still high (I^2^ = 93%, *p* < 0.00001). While all methods detected reduced retinal perfusion in diabetes without diabetic retinopathy (Fig. 4a–c), values for VD% in the superficial capillary plexus (SCP) and assessed in the parafoveal area tended to have the lowest heterogeneity and detected the greatest effect on perfusion (Fig. 4a). Study characteristics are summarised in Table 1.

### Study characteristics of studies included looking at patients with diseases other than diabetes mellitus

Thirty-seven studies assessed VD and FAZ area in patients with diseases other than diabetes (summarised in Table 2), with a similar breadth of assessments as in the diabetes studies in terms of retinal area imaged, vascular layer segmented, and macular region assessed. Also similar to studies of diabetes, VD detected differences more frequently than FAZ area, parafoveal and whole macula VD more frequently than foveal VD and superficial VD more frequently than deep vessel VD.

Patients with Alzheimer’s disease (AD) and mild cognitive impairment (MCI) had reduced foveal, parafoveal, or whole macular perfusion, or increased FAZ area^81,82,84–91,100^, except in one study^83^. Similarly, patients with Parkinson’s disease (PD) had reduced retinal VD and larger FAZ area in two studies^93,94^, and not in one^92^, as did patients with atherosclerosis, stroke and cerebrovascular disease^95,101–103^, and patients with beta thalassaemia^104^, and diabetic patients with CKD^105,106^, and microalbuminurea^107^.

Patients with MS and NMOSD also had lower VD (in the superficial more than the deep retinal circulation) and larger FAZ area^96,97,99^, with patients with a history of optic neuritis having lower retinal perfusion than patients with MS or NMOSD without prior optic neuritis.

Posterior uveitis, including Behcet’s, Vogt-Koyanagi-Harada disease, and Fuch’s heterochromic cyclitis had lower retinal VD and higher FAZ area compared to patients or eyes without uveitis^6,108–116^.

## Discussion

To our knowledge, we present here the first systematic review and meta-analysis of OCTA analysis methods, demonstrating very high heterogeneity of both OCTA analysis methodology employed and reported OCTA data for individual methods. Heterogeneity in analysis methods is demonstrated by the fact that there were more methods of analysing retinal perfusion (197 in this review) than included studies. The included studies varied in perfusion metric used, macula area analysed, equipment manufacturer, and retinal layer segmentation studied, although VD and FAZ area were commonly reported. Although studies investigating the effect of diabetes mellitus on retinal perfusion reporting similar perfusion metrics offered variable and heterogenous results, the OCTA data consistently demonstrated reduced retinal perfusion in patients with diabetes who had, or did not have, diabetic retinopathy when compared to healthy controls.

Reduced retinal perfusion in diabetes and diabetic retinopathy matches the known pathophysiology of the condition^26^, reinforcing the clinical validity of retinal perfusion measurement assessment by OCTA in these patients. However, given that early diabetic retinopathy is associated with retinal ganglion cell (RGC) loss^27^, and RGC degeneration is associated with reduced retinal perfusion^28^, it is not possible to separate primary vascular pathology from changes secondary to neurodegeneration^28^. Further, our meta-analysis demonstrates that not all methods of OCTA analysis reliably detected reductions in retinal perfusion in diabetic retinopathy, suggesting that different approaches have differing sensitivity and reliability. Of the approaches analysed, the superficial vascular plexus (SVP) had the lowest heterogeneity in assessment of retinal perfusion and SVP analysis detected the strongest effects, which has been previously reported in a number of conditions^29^, and is unsurprising given that the superficial retina suffers the fewest noise and projection artefacts^19^, and has the largest blood vessels, with correspondingly higher perfusion^30^ and greater potential for regulation of changes in perfusion^30^. Similarly, retinal perfusion is highest in the parafoveal area, consistent with perfusion assessment in this region detecting the greatest changes.

There are growing calls to standardise OCTA methodology^16,31,32^. We previously compared different OCTA analysis methods, and they concluded that the high variability between metrics and software meant that the different approaches were often not analogous^15^, but that VD data was the most reproducible across platforms and should be reported in OCTA studies, potentially being preferred to skeletonized metrics in the absence of software standardization. It is therefore encouraging that VD was most frequently reported in this study, although heterogeneity was still high. A different study by Rabiolo et al*.*^33^ compared different OCTA algorithms and found that, while the different algorithms all identified important differences between healthy and affected eyes, absolute values were not comparable. This lack of between-platform comparability limits the wider potential of population-level OCTA data. One meta-analysis of fractal dimension perfusion metrics^34^ found that heterogeneity due to different analysis methods limited comparisons, similar to our findings, and again supports calls for standardisation in OCTA protocols. In our meta-analyses we saw high levels of heterogeneity at both the macroscopic level and when analysing different individual analysis methods. One explanation for high individual analysis heterogeneity is the variety of OCTA devices used from different manufacturers that implement different algorithms to determine blood flow or segment vascular layers. A standardised method of quantifying Heidelberg OCTA of the macula and peripapillary vessels has been proposed^35^, although uptake may be limited when manufacturers’ own proprietary algorithms are available^15^.

Limitations of this study include the heterogeneity in reported OCTA methods, which limited synthesis and comparison of analysis methods and findings across different disease states, but highlights the need for standardisation. VD and PD were often used interchangeably and occasionally not defined. The definitions we include in the introduction were the most common definitions in our included studies. Due to high levels of heterogeneity, it was not possible to reliably meet the initial study aims of determining the most sensitive method of OCTA analysis. There were also no papers that studied the test–retest variability of OCTA. Papers did not routinely report reliability, stability, sensitivity, or specificity data for OCTA analyses, which are crucial for test evaluation approaches to the clinical application of OCTA. Finally, while we report increased FAZ area and decreased VD percentages in both patients with diabetes and with diabetic eye disease compared to healthy controls, we recognise that the breadth of the confidence intervals suggest uncertainty about the exact magnitude of this difference.

Currently, there are no standardised reporting guidelines for studies using OCTA—in contrast to the APOSTEL guidelines for OCT studies^36^—and the many thousands of possible OCTA analysis methods available limit reliable comparison of data. To ensure valid comparison of OCTA study results and robust definition of disease characteristics in which retinal perfusion is impaired, we support the suggestion that reporting guidelines and standardisation are urgently required. This would allow consistent reporting to support development of OCTA to its full potential as a ubiquitous clinical imaging modality, similar to OCT, rather than the research tool that it often remains at present^37^. As an initial step, we suggest reporting VD in the parafoveal area and FAZ area as a minimum dataset.

## Conclusion

Analysis and reporting of retinal perfusion using OCTA is highly heterogenous, meaning that despite the myriad of published papers assessing retinal perfusion across different diseases, few direct comparisons can be made. In addition, the stability and reliability of OCTA analyses has been under-studied. We strongly support the need for standardisation of methodology along with OCTA reporting guidelines, and suggest that a minimum dataset for OCTA reporting should include parafoveal VD and FAZ area.

### Supplementary Information

Supplementary Information.

## Data Availability

The datasets used and/or analysed during the current study available from the corresponding author on reasonable request.

## Abbreviations

OCT: Optical coherence tomography
OCTA: Optical coherence tomography angiography
FFA: Fundus fluorescein angiography
PD: Perfusion density
VLD: Vessel length density
FD: Fractal dimension
FAZ: Foveal avascular zone
VD: Vessel density
MEDLINE: Medical Literature Analysis and Retrieval System Online
EMBASE: Excerpta Medica database
PRISMA: Preferred reporting items for systematic reviews and meta-analysis protocols
SVD: Skeletonised vessel density
SFD: Skeletonised fractal dimension
NIH: National Institutes of Health
VD%: Percentage vessel density
DCP: Deep capillary plexus
SCP: Superficial capillary plexus
RGC: Retinal ganglion cell
SVP: Superficial vascular plexus
DM: Diabetes mellitus
NDR: No diabetic retinopathy
Cross-sec: Cross-sectional
SRL: Superficial retinal layer
HD-OCT: High-definition optical coherence tomography
NPDR: Non-proliferative diabetic retinopathy
DRL: Deep retinal layer
T1DM: Type 1 diabetes mellitus
T2DM: Type 2 diabetes mellitus
PDR: Proliferative diabetic retinopathy
SRVP: Superficial retinal vascular plexus
DRVP: Deep retinal vascular plexus
DME: Diabetic macular oedema
GDM: Gestational diabetes mellitus
AD: Alzheimer's disease
MCI: Mild cognitive impairment
POAG: Primary open angle glaucoma
PD: Parkinson’s disease
iRBD: Idiopathic rapid-eye-movement sleep behaviour disorder
NMOSD-ON: Neuromyelitis optica spectra disorder without optic neuritis
NMOSD + ON: Neuromyelitis optica spectra disorder with optic neuritis
MS-ON: Multiple sclerosis without optic neuritis
MS + ON: Multiple sclerosis with optic neuritis
aMCI: Amnestic mild cognitive impairment
CSVD: Cerebral small vessel disease
CKD: Chronic kidney disorder
VKHD + SGF: Vogt–Koyanagi–Harada disease with sunset glow fundus
VKHD-SGF: Vogt–Koyanagi–Harada disease without sunset glow fundus
VKHD: Vogt–Koyanagi–Harada disease
SD-OCT: Spectral-domain optical coherence tomography
SS-OCT: Swept-source optical coherence tomography
OMAG: Optical microangiography
SS-ADA: Split-spectrum amplitude decorrelation angiography
OCTARA: Optical coherence tomography angiography ratio analysis
CODAA: Complex optical coherence tomography signal difference analysis angiography
FSPA: Full spectrum probabilistic approach
NFL: Nerve fibre layer
GCL: Ganglion cell layer
IPL: Inner plexiform layer
INL: Inner nuclear layer
ONL: Outer nuclear layer
DVP: Deep vascular plexus
SCC: Superficial capillary complex
DCC: Deep capillary complex
