# Supplementary material for: Optical coherence tomography angiography analysis methods: a systematic review and meta-analysis
Source: Sci Rep. 2024 Apr 26;14:9643. doi: 10.1038/s41598-024-54306-3 (PMC11053039; doi:10.1038/s41598-024-54306-3)
Supplement: Supplementary file 1 — Supplementary Information. [file 41598_2024_54306_MOESM1_ESM.pdf]

Optical coherence tomography angiography analysis methods: a systematic review and meta-analysis

Ella Courtie[1, 2, 3]*, James Kirkpatrick[4]*, Matthew Taylor[5, 6, 7], Livia Faes[8], Xiaoxuan Liu[5, 6, 9], Ann Logan[10, 11], Tonny Veenith[1, 12, 13], Alastair K. Denniston[2, 8, 9], Richard J. Blanch[1, 2, 3, 14]

1. Neuroscience and Ophthalmology Research Group, University of Birmingham, Birmingham, UK.

2. Department of Ophthalmology, Queen Elizabeth Hospital Birmingham, University Hospitals Birmingham NHS Foundation Trust, West Midlands, UK.

3. Surgical Reconstruction and Microbiology Research Centre, University Hospitals Birmingham NHS Foundation Trust, Birmingham, UK.

4. 1 Armoured Medical Regiment, British Army, Bhurtpore Barracks, Tidworth, UK.

5. University Hospitals Birmingham NHS Foundation Trust, Birmingham, UK

6. University of Birmingham, Birmingham, UK.

7. Birmingham Women's and Children's NHS Foundation Trust, Birmingham, UK

8. NIHR Biomedical Research Centre at Moorfields Eye Hospital NHS Foundation Trust–UCL Institute of Ophthalmology, London, UK

9. NIHR Birmingham Biomedical Research Centre, University Hospitals Birmingham NHSFT, Birmingham UK.

10. Axolotl Consulting Ltd., Worcestershire, Droitwich, UK.

11. Division of Biomedical Sciences, Warwick Medical School, University of Warwick, UK.

12. Critical Care Unit, Queen Elizabeth Hospital Birmingham, University Hospitals Birmingham NHS Foundation Trust, Birmingham, UK.

13. Department of Trauma Sciences, University of Birmingham, Birmingham, UK.

14. Academic Department of Military Surgery and Trauma, Royal Centre for Defence Medicine, Birmingham, UK.

*EC and JK are joint first authors.

Supplementary Figure 1. Heidelberg Spectralis representative optical coherence tomography (OCT) and OCT angiography (OCTA) images and segmentation: 1a) En face infra-red view of the macula (left panel) and cross-sectional OCT image through the fovea (right panel), with the internal limiting membrane (ILM), nerve fibre layer (NFL), ganglion cell layer (GCL), inner plexiform layer (IPL), inner nuclear layer (INL), outer plexiform layer (OPL) and retinal pigment epithelium (RPE) labelled. 1b) OCTA superficial vascular plexus displayed en face. 1c) OCTA deep capillary plexus displayed en face. The FAZ has been manually outlined in both 1b and 1c to demonstrate the FAZ area calculated from OCTA images. Vessel density percentage is calculated by measuring white pixel density in comparison with black pixel density.

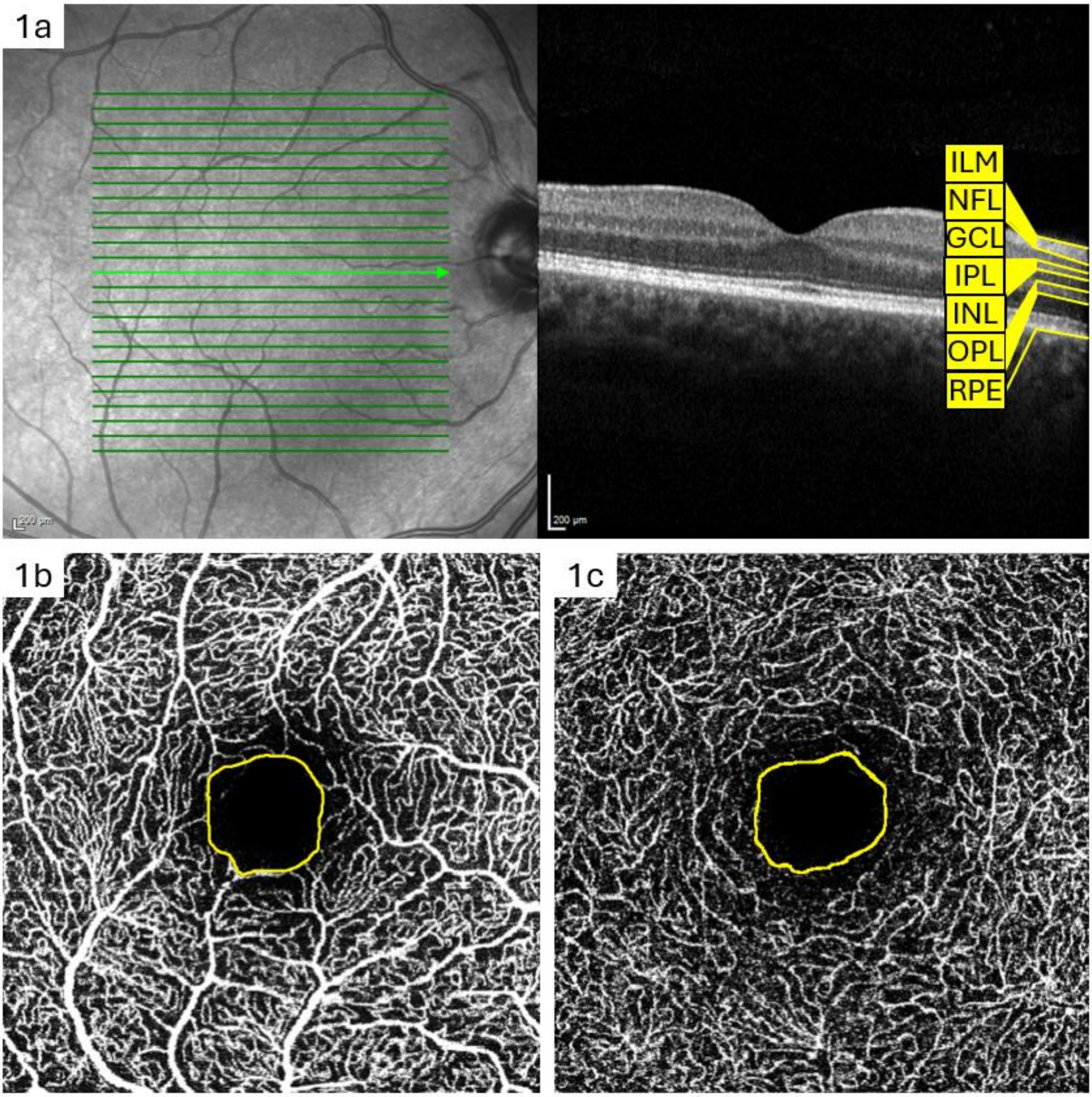

Supplementary Figure 2. Applied search strategy

Patient/problem: individuals who have had an OCTA scan and include either normal patients only or normal patients compared to patients with a disease state.

Intervention: methods of OCTA analysis used and their mean and SD effect size (sensitivity to change)

Comparisons:

1) normal vs disease condition
2) normal patients intra (repeated measures) and inter (other normal patients) subject variability.

Outcome:

1) sensitivity to detect disease states
2) intra and intersubject agreement / variability

Search Strategy:

1. "Optical coherence tomography angiography".mp or exp optical coherence tomography/ or exp optical coherence tomography angiography
2. OCTA.mp
3. 1 or 2
4. Macula* retina.mp or exp retina/
5. Sepsis.mp or exp sepsis/
6. Diabetes.mp
7. Uveitis.mp or exp uveitis/
8. Hypotension.mp or exp hypotension/
9. Neurodegenerative disorders.mp
10. Multiple sclerosis.mp or exp multiple sclerosis/
11. Cerebrovascular disease.mp or cerebrovascular disease/
12. Stroke.mp
13. Mild cognitive impairment.mp or exp mild cognitive impairment/
14. Dementia.mp or exp dementia/
15. Alzheimer* Disease.mp or exp Alzheimer disease/
16. Parkinson* Disease.mp or exp Parkinson disease/
17. 5 or 6 or 7 or 8 or 9 or 10 or 11 or 12 or 13 or 14 or 15 or 16
18. 3 and 4 and 17

Supplementary Table 1. Inclusion and exclusion criteria for literature searches

Study criteria	Inclusion	Exclusion
Study design	• Any studies are included which involves using OCTA analysis to measure global perfusion of the retina in individuals who: (1) are healthy, (2) have a retinal disorder due to a systemic disease e.g., diabetes, or (3) have retinal changes due to a neurodegenerative disorder e.g., Alzheimer's disease.<br>• Published between 2010:2022.	• Studies which are not written in English.<br>• Retrospective study
Population	• Human studies.<br>• No age restrictions.<br>• One or both eyes included.	• Retinal disorders which cause focal anatomical change e.g., AMD.<br>• Animal studies.
Study methodology	• Studies that include patients having OCTA scans which are analysed by any of the following (skeletonised or binarized): perfusion density; fractal dimension; vessel length density; FAZ area or perimeter.<br>• Studies looking at foveal, parafoveal and whole areas of the macula<br>AND<br>EITHER<br>- Group 1: studies comparing findings in normal patients with pathology.<br>OR<br>- Group 2: Studies that include patients having repeated OCTA scans over time.	• Studies with no comparison group.<br>• Studies using only OCT scans.<br>• Studies looking at retinal blood flow using methods other than OCTA e.g., fluorescein angiography.<br>• Studies investigating choroid/choriocapillaris/peripapillary blood flow.<br>• Studies with <50 participants.<br>• Case reports<br>• Reviews/systematic review/meta-analysis.<br>• Abstracts.

Supplementary Table 2. Papers excluded during screening (n=103)

Authors	Title	Reason
**Abraham**	Cognitive decline in older adults: What can we learn from optical coherence tomography (OCT)-based retinal vascular imaging?	Data not clear if disease or normal. Data not in useable format
**Akil, H**	Corneal confocal microscopy and not optical coherence tomography detects progressive worsening of neuropathy in Type 1 Diabetes	Abstract only
**Alibhai, A**	Quantification of retinal capillary nonperfusion in diabetics using wide-field optical coherence tomography angiography	Studied non-perfusion
**Aly, L**	Optical coherence tomography angiography indicates subclinical retinal disease in neuromyelitis optica spectrum disorders	Duplicate
**Arnould, L**	The EYE-MI pilot study: A prospective acute coronary syndrome cohort evaluated with retinal optical coherence tomography angiography	No standard deviation values, wrong area of macula analysed
**Arrigo, A**	Octa-based identification of different vascular patterns in stargardt disease	Retinal structural disease
**Asanad, S**	Retinal thickness and vascular pathology as ocular biomarkers for schizophrenia: Morphometric analysis of the peripapillary and macular regions using OCT and OCTA in vivo	Abstract only
**Ashraf, M**	Statistical model of optical coherence tomography angiography parameters that correlate with severity of diabetic retinopathy	Disease only, no control group
**Ashraf, M**	Interaction between the Distribution of Diabetic Retinopathy Lesions and the Association of Optical Coherence Tomography Angiography Scans with Diabetic Retinopathy Severity	Disease only, no control group and retrospective
**Barsukov, A**	The relationship between the indicators of the retina condition and other target organ changes in uncomplicated essential hypertension	Foreign language
**Ceravolo, I**	Foveal avascular zone analysis by Coherence tomography angiography in Patients with DM1 and DM2 with and without Diabetic Retinopathy	Abstract only
**Chen, Q**	Macular vascular fractal dimension in the deep capillary layer as an early indicator of microvascular loss for retinopathy in type 2 diabetic patients	Retrospective
**Choi, W**	Ultrahigh speed swept source optical coherence tomography angiography of retinal and choriocapillaris alterations in diabetic patients with and without retinopathy	Prototype OCTA scanner used
**Comez, A**	Quantitative analysis of retinal microcirculation in optical coherence tomography angiography in cases with Behcet's disease without ocular involvement	Study population too small
**Conti, F**	Choriocapillaris and retinal vascular plexus density of diabetic eyes using split-spectrum amplitude decorrelation spectral-domain optical coherence tomography angiography	Retrospective
**Czako, C**	Evaluation of diabetic microangiopathy using optical coherence tomography angiography	Foreign language
**Dimitrova, G**	Quantitative retinal optical coherence tomography angiography in patients with diabetes without diabetic retinopathy	Scan type not mentioned

**Ebihara, S**	Relationships between the vascular structure and neural function of the macula in patients with diabetes mellitus	Data values not specified, graphs only
**Farci, R**	Optical coherence tomography angiography in multiple sclerosis: A cross-sectional study	Data values not specified, graphs only
**Fayed, A**	Optical coherence tomography angiography reveals progressive worsening of retinal vascular geometry in diabetic retinopathy and improved geometry after panretinal photocoagulation	Data values not specified, graphs only
**Feng, L**	Macular vascular density changes following cataract surgery in diabetic patients: An optical coherence tomography angiography study	Area of macula analysed not clear
**Fickweiler, W**	Association of cognitive function with retinal neural structure and vasculature in type 1 diabetes	Data values not specified, graphs only
**Frizziero, L**	Early retinal changes by oct angiography and multifocal electroretinography in diabetes	Data values not specified, graphs only
**Ghassemi**	Quantitative assessment of vascular density in diabetic retinopathy subtypes with optical coherence tomography angiography	No standard deviation values
**Govindasamy, N**	Artificial intelligence effectively combined OCT and OCTA indices to improve early detection of diabetic retinopathy (DR)	Abstract only
**Govindaswamy, N**	Vascular changes precede tomographic changes in diabetic eyes without retinopathy and improve artificial intelligence diagnostics	No standard deviation values, wrong area of macula analysed
**Gregory Yu, M**	Cardiac and retinal imaging studies suggest comparable protective factors between cardiovascular disease and retinopathy in long duration type 1 diabetes	Abstract only
**Goker, Y**	Quantitative Analysis of Optical Coherence Tomography Angiography Features in Patients with Nonocular Behcet's Disease.	Study population too small
**Guo, J**	Study on the correlation between tear film lipid layer thickness and macular microvascular changes in patients with diabetic retinopathy	Abstract only
**Hirano, T**	Quantifying vascular density and morphology using different swept-source optical coherence tomography angiographic scan patterns in diabetic retinopathy	Study population too small
**Hogg, R**	Early functional and imaging markers of retinal dysfunction prior to onset of clinically detectable diabetic retinopathy (DR)	Abstract only
**Hsiao, C**	Correlation of retinal vascular perfusion density with dark adaptation in diabetic retinopathy	Retrospective
**Hsiao, C**	The correlation of retinal vascular perfusion density and dark adaptation in diabetic retinopathy	Duplicate
**Jiang, H**	Visual Function and Disability Are Associated with Increased Retinal Volumetric Vessel Density in Patients with Multiple Sclerosis	Data values not specified, graphs only
**Jiang, H**	Altered macular microvasculature in mild cognitive impairment and Alzheimer disease	Data values not specified, graphs only
**Kaizu, Y**	Flow Density in Optical Coherence Tomography Angiography is Useful for Retinopathy Diagnosis in Diabetic Patients	Study population subsets not defined
**Kaizu, Y**	Optical Coherence Tomography Angiography Reveals Spatial Bias of Macular Capillary Dropout in Diabetic Retinopathy.	Wrong area of macula analysed

**Karakucuk, Y**	Evaluation of the effect of fingolimod (FTY720) on macular perfusion by swept-source optical coherence tomography angiography in patients with multiple sclerosis.	Wrong area of macula analysed
**Kasumovic, A**	Assessment of Retinal Microangiopathy in Chronic Kidney Disease Patients	Data values not specified, graphs only
**Kasumovic, A**	Optical coherence tomography angiography contributions in classification of nonproliferative diabetic retinopathy	Data values not specified, graphs only. No standard deviation values
**Kim, K**	Optical coherence tomography angiography analysis of foveal microvascular changes and inner retinal layer thinning in patients with diabetes	Data values not specified-presented as correlation coefficients
**Koca, S**	Comparison of Optical Coherence Tomography Angiography Findings in Patients with Behcet's Disease and Healthy Controls	No standard deviation values
**Kuonen, A**	Perifoveal capillary changes in diabetic patients and association between severity and type of diabetes, visual acuity, and enlargement of non-flow area in the retinal capillary plexuses	No standard deviation values
**Kwapong, W**	Retinal microvascular impairment in the early stages of Parkinson's disease	Wrong area of macula analysed
**Lanzillo, R**	Retinal vascular density in multiple sclerosis: a 1-year follow-up.	Disease only, no control group
**Laotaweerungsawat, S**	OCT Angiography Assessment of Retinal Microvascular Changes in Diabetic Eyes in an Urban Safety-Net Hospital	Data values not specified, graphs only
**Lee, G**	Differential patterns of parafoveal and peripapillary vessel density in multiple sclerosis and neuromyelitis optica spectrum disorder	Retrospective
**Lee, M**	Effects of prolonged type 2 diabetes on the inner retinal layer and macular microvasculature: An optical coherence tomography angiography study	Retrospective
**Lee, T**	Relationship between N95 amplitude of pattern electroretinogram and optical coherence tomography angiography in open-angle glaucoma	Wrong area of macula analysed
**Lei, J**	Distinctive Analysis of Macular Superficial Capillaries and Large Vessels Using Optical Coherence Tomographic Angiography in Healthy and Diabetic Eyes.	Wrong area of macula analysed
**Li, F**	Association of foveal avascular zone area withstructural and functional progression in glaucoma patients	Disease only, no control group
**Li, X**	Identifying Microvascular and Neural Parameters Related to the Severity of Diabetic Retinopathy Using Optical Coherence Tomography Angiography.	Disease only, no control group
**Li, Z**	Do microvascular changes occur preceding neural impairment in early-stage diabetic retinopathy? Evidence based on the optic nerve head using optical coherence tomography angiography	Optic disc analysed only
**Li, Z**	Optical coherence tomography angiography findings of neurovascular changes in type 2 diabetes mellitus patients without clinical diabetic retinopathy	Unclear scan size
**Liu, C**	Optical coherence tomography angiography helps distinguish multiple sclerosis from AQP4-	Data values not specified, graphs only

	IgG-seropositive neuromyelitis optica spectrum disorder	
**Liu, L**	Peripapillary region perfusion and retinal nerve fiber layer thickness abnormalities in diabetic retinopathy assessed by OCT angiography	Optic disc analysed only
**Liu, Y**	Morphological changes in and quantitative analysis of macular retinal microvasculature by optical coherence tomography angiography in hypertensive retinopathy	Retrospective
**Loba, M**	Optical coherence tomography angiography - Novel method to access retinal microcirculation in patients with diabetic retinopathy	Not appropriate for analysis
**Lommatzsch, C**	OCTA vessel density changes in the macular zone in glaucomatous eyes	Unclear which instrument used
**Magera, L**	CHANGES OF THE FOVEAL AVASCULAR ZONE AND MACULAR MICROVASCULATURE WITHIN THE FRAMEWORK OF OCT ANGIOGRAPHY EXAMINATION IN YOUNG PATIENTS WITH TYPE 1 DIABETES (PILOT STUDY).	Unclear which instrument used
**Magera, L**	ZMĚNY FOVEALNI AVASKULARNI ZONY A MAKULARNI MIKROVASKULATURY V RAMCI VYSETŘENI OCT ANGIOGRAFIE U MLADYCH DIABETIKŮ 1. TYPU (PILOTNI STUDIE), CHANGES OF THE FOVEAL AVASCULAR ZONE AND MACULAR MICROVASCULATURE WITHIN THE FRAMEWORK OF OCT ANGIOGRAPHY EXAMINAT	Duplicate in different language
**Mameli, C**	Analysis of Retinal Perfusion in Children, Adolescents, and Young Adults with Type 1 Diabetes Using Optical Coherence Tomography Angiography	No standard deviation values
**Marques, I**	Different retinopathy phenotypes in type 2 diabetes predict retinopathy progression.	Data values not specified, graphs only
**Mastropasqua, R**	Widefield optical coherence tomography angiography in diabetic retinopathy.	Wrong area of macula analysed
**Murueta-Goyena, A**	Foveal Remodeling of Retinal Microvasculature in Parkinson's Disease	Unclear scan size
**Murphy, O**	Alterations in the retinal vasculature occur in multiple sclerosis and exhibit novel correlations with disability and visual function measures	Not appropriate for analysis
**Murphy, O**	Optical Coherence Tomography and Optical Coherence Tomography Angiography Findings After Optic Neuritis in Multiple Sclerosis	Data values not specified, graphs only
**Ong, J**	Macrophage-like cell density is increased in proliferative diabetic retinopathy characterized by optical coherence tomography angiography	Not appropriate for analysis
**Pascual-Prieto, J**	Utility of optical coherence tomography angiography in detecting vascular retinal damage caused by arterial hypertension	Wrong area of macula analysed
**Peng, C**	Structural and Microvascular Changes in the Macular Are Associated With Severity of White Matter Lesions	Disease only, no control group
**Pierro, L**	Quantitative Optical Coherence Tomography Angiography Detects Retinal Perfusion Changes in Carotid Artery Stenosis	Area of macula analysed not clear
**Querques, G**	Functional and morphological changes of the retinal vessels in Alzheimer's disease and mild cognitive impairment.	Area of macula analysed not clear

**Ra H**	Discordance in Retinal and Choroidal Vascular Densities in Patients with Type 2 Diabetes Mellitus on Optical Coherence Tomography Angiography	Data values not specified, graphs only
**Rodrigues, T**	Macular OCT-angiography parameters to predict the clinical stage of nonproliferative diabetic retinopathy: an exploratory analysis	Disease only, no control group
**Salobrar-García, E**	Changes in visual function and retinal structure in the progression of Alzheimer's disease	Data values not specified, graphs only
**Shah, Manan**	Use of optical coherence tomography angiography in evaluating foveal vascularization in Type 1 Diabetes Mellitus	Abstract only
**Shaw, Lincoln T**	Quantitative optical coherence tomography angiography (Octa) parameters in a black diabetic population and correlations with systemic diseases	Disease only, no control group
**Shaw, Lincoln T**	Quantitative optical coherence tomography angiography (Octa) parameters in a black diabetic population and correlations with systemic diseases	Disease only, no control group
**Santos, T**	Swept-source OCTA quantification of capillary closure predicts ETDRS severity staging of NPDR	Not appropriate for analysis
**Shi, C**	Characterization by fractal dimension analysis of the retinal capillary network in parkinson disease	Wrong area of the macula analysed
**Shin, Joo Young**	Changes in retinal microvasculature and retinal layer thickness in association with apolipoprotein E genotype in Alzheimer's disease	Area of macula analysed not clear
**Shu wei Ting, D**	Optical coherence tomographic angiography in type 2 diabetes and diabetic retinopathy	Disease only, no control group
**Silverstein, S**	Retinal microvasculature in schizophrenia	Not appropriate for analysis
**Sun, C**	Systemic hypertension associated retinal microvascular changes can be detected with optical coherence tomography angiography	Retrospective
**Szkodny, D**	Retinal oct findings in patients after covid infection	No standard deviation values
**Tan, B**	Quantitative Microvascular Analysis with Wide-Field Optical Coherence Tomography Angiography in Eyes with Diabetic Retinopathy	No- vessel layer not seperated
**Tang, F**	Progressive Changes in Microvasculature in Eyes with Different Diabetic Retinopathy Severity: A Longitudinal Study	Abstract only
**Tang, F**	Relationship of intercapillary area with visual acuity in diabetes mellitus: An optical coherence tomography angiography study	Data values not specified- presented as correlation coefficients
**Turkcu, F**	Automated quantification of foveal avascular zone and vascular density in Behcet's disease	Retrospective
**Van Bentum, R**	Microvascular changes of the retina in ankylosing spondylitis, and the association with cardiovascular disease - the eye for a heart study	No- area of macula not clear
**Vujosevic, S**	Early Detection of Microvascular Changes in Patients with Diabetes Mellitus without and with Diabetic Retinopathy: Comparison between Different Swept-Source OCT-A Instruments.	Not all data values available
**Wang, Han-Ying**	Detection of vessel density changes in eyes of patients with diabetic retinopathy and diabetic	Foreign language

	macular edema using optical coherence tomography angiography	
**Wang, J**	Retinal and Choroidal vascular changes in coronary heart disease: An optical coherence tomography angiography study	Data values not specified, graphs only
**Wang, Q**	Albuminuria and retinal vessel density in diabetes without diabetic retinopathy: the Kailuan Eye Study	Disease only, no control group
**Wintergerst, M**	Optical Coherence Tomography Angiography in Intermediate Uveitis	Study population too small
**Xiang, X**	Clinical application of OCTA in observation of macular blood flow density in patients with diabetic retinopathy	Foreign language
**Xie, Ke-Ren**	Evaluation of retinal blood flow changes in patients with type 1 diabetes mellitus by optical coherence tomography angiography	Foreign language
**Xie, N**	Macular vessel density in diabetes and diabetic retinopathy with swept-source optical coherence tomography angiography	Data values not specified, graphs only
**You, Q**	Macular vessel density measured with optical coherence tomography angiography and its associations in a large population-based study	No comparison group
**You, Q**	Optical Coherence Tomography Angiography Avascular Area Association With 1-Year Treatment Requirement and Disease Progression in Diabetic Retinopathy.	Disease only, no control group. Data values not specified, graphs only
**Zeng, Y**	Changes of macular vessel density and structures in different early stages of diabetic retinopathy	Foreign language
**Zhu, Z**	Clinical effect of conbercept on improving diabetic macular ischemia by OCT angiography	Disease only, no control group
**Zhuang, X**	Associations between retinal microvasculature/microstructure and renal function in type 2 diabetes patients with early chronic kidney disease	Retrospective

Supplementary Table 3. Risk of bias table using the National Institutes of Health (NIH) quality assessment tool for observational cohort and cross-sectional studies and the NIH quality assessment tool of case-control study.

		NIH Quality assessment tool questions														
		1	2	3	4	5	6	7	8	9	10	11	12	13	14	
		Research question clearly defined/stated	Study population clearly defined	Participation rate >50%	Groups recruited from the same population and uniform eligibility criteria	Sample size justification included	Exposure assessed prior to outcome measurement	Sufficient timeframe to see an effect	Different level of the exposure of interest	Exposure measurements clearly defined, valid and reliable	Repeated exposure assessment	Outcome measures clearly defined, valid and reliable	Blinding of outcome assessors	Was loss to follow-up 20% or less	Were confounding variables measured and adjusted for statistically	Cross-sectional and cohort
		1	2	3	4	5	6	7	8	9	10	11	12			
		Research question clearly defined/stated	Study population clearly defined	Sample size justification included	Controls selected from similar population as cases	Inclusion and exclusion criteria prespecified and applied uniformly	Cases clearly defined and differentiated from controls	Random selection of study participants	Concurrent controls	Exposure assessed prior to outcome measurement	Exposure measures and assessment	Blinding of exposure assessors	Were confounding variables measured and adjusted for statistically			Case-control
Paper	Study Design															Quality
Agarwal, 2021	Cross-sec			NR									NR	NA		Poor
Agra, 2021	Cross-sec			NR									NR	NA		Fair
Aksoy, 2020 (a)	Cross-sec			NR									NR	NA	NR	Poor
Aksoy 2020 (b)	Cross-sec			NR									NR	NA	NR	Poor
Aly, 2021	Cross-sec			NR									NR	NA		Good
Aschauer, 2020	Cohort			NR									NR			Good
Bhanushali, 2016	Cross-sec			NR									NR	NA	NR	Poor
Bontzos, 2021	Cross-sec			NR									NR	NA		Good
Buyuktepe, 2020	Cross-sec			NR									NR	NA	NR	Fair
Bulut, 2018	Cross-sec			NR		NR							NR	NA	NR	Poor
Cankurtaran, 2020	Cross-sec			NR									NR	NA		Fair
Cao, 2018	Cross-sec			NR									NR	NA	NR	Fair
Carnevali, 2017	Cross-sec			NR	CD					CD			NR	NA		Poor
Choi, 2020	Cross-sec			NR									NR	NA		Fair
Chua, 2020	Cross-sec			NR									NR	NA		Fair
Ciloglu, 2019	Cross-sec			NR									NR	NA	NR	Poor
Cinar, 2020	Cross-sec			NR									NR	NA		Fair
Cordon, 2020	Cross-sec			NR	CD								NR	NA	NR	Poor
Criscuolo, 2020	Cross-sec			NR									NR	NA		Fair
Czako, 2019	Cross-sec			NR									NR	NA		Fair
de Carlo, 2015	Cross-sec			NR										NA	CD	Fair
Değirmenci, 2020	Cross-sec			NR									NR	NA	NR	Poor
Demir, 2020	Cross-sec			NR									NR	NA		Fair
Durbin, 2017	Cross-sec			NR									NR	NA		Fair
Fan, 2021	Cross-sec			NR										NA		Good
Forte, 2020	Cross-sec			NR									NR	NA		Fair
Furino, 2020	Cross-sec			NR									NR	NA		Fair
Golebiewska, 2017	Cross-sec			NR									NR	NA		Fair
Haan, 2019	Cross-sec			NR										NA		Good
Hirano, 2019	Cross-sec			NR									NR	NA		Fair
Inanc, 2019	Cross-sec			NR									NR	NA		Fair
Kara, 2021	Cross-sec			NR									NR	NA		Fair

Karaca, 2020	Cross-sec			NR										NA	NR	Poor
Karaküçük, 2020	Cross-sec			NR									NR	NA	NR	Poor
Karst, 2020	Cross-sec			NR									NR	NA		Good
Kazanci, 2020	Cross-sec			NR									NR	NA		Poor
Kim, 2016	Cross-sec			NR									NR	NA		Good
Kim, 2020	Cohort			NR				CD					NR	NA		Good
Koçer, 2021	Cross-sec			NR									NR	NA		Fair
Lahme, 2018	Cross-sec			NR	NR			NR					NR	NA		Fair
Lanzillo, 2019	Cohort			NR									NR	NA	NR	Good
Li Rudvan, 2019	Cross-sec			NR									NR	NA		Good
Li, h, 2021	Cross-sec			NR									NR	NA	NR	Fair
Li, t, 2019	Cross-sec			NR									NR	NA		Fair
Liu, b, 2021	Cross-sec			NR	CD								NR	NA		Fair
Liu, g, 2021	Cross-sec			NR									NR	NA		Fair
Marques, 2021	Cohort			NR										NR		Good
Meshi, 2019	Case-control							NA	NR		CD					Good
Niestrata-Ortiz, 2019 (a)	Cross-sec			NR										NA		Fair
Niestrata-Ortiz, 2019 (b)	Cross-sec			NR	CD									NA	CD	Fair
Oliverio, 2021	Cross-sec			NR										NA		Good
Peng, 2020	Case-control				NR			NR	NR			NR				Fair
Rascuna, 2020	Cross-sec			NR									NR	NA		Poor
Robbins, 2021	Cross-sec			NR									NR	NA		Fair
Robbins, 2021	Cross-sec			NR									NR	NA		Poor
Ryu, 2021	Cross-sec			NR									NR	NA		Fair
Sacconi, 2019	Cross-sec			NR									NR	NA		Fair
Shen, 2019	Cross-sec			NR									NR	NA		Fair
Shin, 2021	Cross-sec							NA	NR			NR				Good
Shoji, 2022	Cohort			NR										NA		Good
Simonett, 2017	Cross-sec			NR									NR	NA		Fair
Smid, 2021	Cross-sec			NR									NR	NA		Fair
Somilleda-Ventura, 2019	Cross-sec			NR									NR	NA	CD	Poor
Stulova, 2021	Case-control				NR	CD		NA	NR			NR	CD			Poor
Sugimoto, 2019	Cross-sec			NR									NR	NA		Good
Sun, 2019	Cohort			NR									NR			Good
Tarek, 2021	Case-control							NA					CD			Good
Tian, 2019	Cross-sec			NR										NA		Good
Toto, 2017	Cross-sec			NR	NR									NA		Fair
Veiby, 2020	Cross-sec		CD	NR										NA		Good
Vujosevic, 2018	Cross-sec			NR										NA		Fair
Vujosevic 2020	Cross-sec			NR									NR	NA		Fair
Wang w, 2020	Cross-sec			NR										NA		Good
Wang x, 2021	Cross-sec			NR										NA		Good
Wang x, 2021	Cross-sec			NR									NR	NA		Fair
Wu, 2020	Cross-sec			NR									NR	NA		Fair
Yan, 2021	Cross-sec			NR				NR					NR	NA		Fair
Yang, 2020	Cross-sec			NR									NR	NA		Fair
Yilmaz h, 2020	Cross-sec			NR									NR	NA		Fair
Yilmaz p, 2021	Cross-sec			NR									NR	NA	NR	Fair
Yoon, 2017	Cross-sec			NR									NR	NA		Fair
Zabel, 2019	Cross-sec			NR									NR	NA		Fair
Zabel, 2021	Cross-sec			NR									NR	NA		Fair
Zeng, 2019	Cross-sec			NR	CD								NR	NA	NR	Poor
Zeng, 2020	Cross-sec			NR	CD								NR	NA		Poor
Zhang x, 2020	Cross-sec			NR									NR	NA		Fair

Zhang y, 2020	Cross-sec			NR										NA		Good
Zou, 2020	Cross-sec			NR									NR	NA		Fair
